# Trefoil factor 3 promotes metastatic seeding and predicts poor survival outcome of patients with mammary carcinoma

**DOI:** 10.1186/s13058-014-0429-3

**Published:** 2014-09-30

**Authors:** Vijay Pandey, Zheng-Sheng Wu, Min Zhang, Rui Li, Jian Zhang, Tao Zhu, Peter E Lobie

**Affiliations:** 1 Cancer Science Institute of Singapore, National University of Singapore, MD6, 11-01, 14 Medical Drive, Singapore, 117599 Singapore; 2 Department of Pharmacology, National University of Singapore, 21 Lower Kent Ridge Road, Singapore, 119077 Singapore; 3 Hefei National Laboratory for Physical Sciences at Microscale and School of Life Sciences, University of Science and Technology of China, 96, Jin Zhai Road, Baohe District, Hefei, 230026 Anhui P.R. China; 4 Department of Pathology, Anhui Medical University, 81 Meishan Road, Shushan district 230032 Hefei, Anhui, P.R. China; 5 Shenzhen Institute of Advanced Technology, Chinese Academy of Science, 1068 Xueyuan Avenue, Shenzhen, 518055 P.R. China; 6 National Cancer Institute of Singapore, National University Health System, 5 Lower Kent Ridge Road, Singapore, 119077 Singapore

## Abstract

**Introduction:**

Recurrence or early metastasis remains the predominant cause of mortality in patients with estrogen receptor positive (ER+) mammary carcinoma (MC). However, the molecular mechanisms underlying the initial progression of ER+ MC to metastasis remains poorly understood. Trefoil factor 3 (TFF3) is an estrogen-responsive oncogene in MC. Herein, we provide evidence for a functional role of TFF3 in metastatic progression of ER+ MC.

**Methods:**

The association of TFF3 expression with clinicopathological parameters and survival outcome in a cohort of MC patients was assessed by immunohistochemistry. The expression of TFF3 in MCF7 and T47D cells was modulated by forced expression or siRNA-mediated depletion of TFF3. mRNA and protein levels were determined using qPCR and western blot. The functional effect of modulation of TFF3 expression in MC cells was determined *in vitro* and *in vivo*. Mechanistic analyses were performed using reporter constructs, modulation of signal transducer and activator of transcription 3 (STAT3) expression, and pharmacological inhibitors against c-SRC and STAT3 activity.

**Results:**

TFF3 protein expression was positively associated with larger tumour size, lymph node metastasis, higher stage, and poor survival outcome. Forced expression of TFF3 in ER+ MC cells stimulated colony scattering, cell adhesion to a Collagen I-coated matrix, colony formation on a Collagen I- or Matrigel-coated matrix, endothelial cell adhesion, and transmigration through an endothelial cell barrier. *In vivo*, forced expression of TFF3 in MCF7 cells stimulated the formation of metastatic nodules in animal lungs. TFF3 regulation of the mRNA levels of epithelial, mesenchymal, and metastatic-related genes in ER+ MC cells were consistent with the altered cell behaviour. Forced expression of TFF3 in ER+ MC cells stimulated phosphorylation of c-SRC that subsequently increased STAT3 activity, which lead to the downregulation of E-cadherin. siRNA-mediated depletion of TFF3 reduced the invasiveness of ER+ MC cells.

**Conclusions:**

TFF3 expression predicts metastasis and poor survival outcome of patients with MC and functionally stimulates cellular invasion and metastasis of ER+ MC cells. Adjuvant functional inhibition of TFF3 may therefore be considered to ameliorate outcome of ER+ MC patients.

**Electronic supplementary material:**

The online version of this article (doi:10.1186/s13058-014-0429-3) contains supplementary material, which is available to authorized users.

## Introduction

Metastatic sequelae are the predominant causes of mortality in patients with mammary carcinoma (MC) [1]. The metastatic spread of carcinoma cells is a complex process involving intravasation, survival in the circulation, extravasation, and colonisation at targeted organs [2]. The risk of distant metastasis and hence relapse of disease is proposed to be largely influenced by underlying molecular determinants and their signaling cascades in MC cells [2]. The estrogen receptor positive (ER+) (luminal A and B [3]) subset constitutes the majority (approximately 60%) of MC and is associated with a more favourable prognosis compared to ER-negative MC due to the availability of effective estrogen antagonists [3]. Even though patients with ER+ MC respond favourably to anti-estrogen treatment, a significant proportion of this subgroup will still develop regional recurrence and associated distant metastasis [4]-[6], after treatment with the standard five-year tamoxifen regimen [7]. Despite the use of aromatase inhibitors and adjuvant chemotherapy in combination with anti-estrogenic agents that may benefit some patients, the significant risk of metastatic recurrence remains [8],[9]. Currently, the molecular determinants for metastatic relapse and the underlying mechanisms, knowledge of which is essential for effective therapeutic intervention, requires further delineation.

Trefoil factor 3 (TFF3) is an estrogen-responsive gene, and its expression level is positively correlated with ER+ status in MC [10],[11]. Elevated levels of TFF3 expression have also been reported in the molecular apocrine subtype of ER-negative MC, characterised by the expression of androgen receptor (AR), FOXA1, and a high frequency of human epidermal growth factor receptor type 2 (HER2) expression [12],[13]. In MC, TFF3 behaves as an oncogene, the forced expression of which promotes MC cell proliferation, survival, oncogenicity and invasion [14]. In addition, tumour expression of *TFF3* mRNA predicts a worse survival outcome in ER+ patients treated with tamoxifen and increased expression of TFF3 both reduces sensitivity of ER+ MC cells to tamoxifen and mediates acquired resistance to tamoxifen [14]. Inhibition or depletion of TFF3 in tamoxifen-resistant MC cells is able to restore tamoxifen sensitivity [14]. A number of reports indicate an association between TFF3 expression and MC metastasis. For example, higher expression of TFF3 was observed in invasive ductal carcinoma [15]; TFF3 expression was observed to be associated with the localization of metastatic MC cells to bone and to micrometastatic MC [16],[17]; TFF3 was included in a panel of four genes that specifically detected minimal residual disease in the circulation and consequently predicted worse survival in patients with metastatic MC [17]; and TFF3 has been used as a marker for the detection of disseminated MC cells together with TFF1 [18]. A recent histopathological analysis has also demonstrated that elevated expression of TFF3 is associated with muscle, neural, and lymphovascular invasion of MC [11]. In that study, TFF3 expression was identified as an independent, predictive marker of lymphovascular invasion and lymph node involvement in MC [11]. Although we have previously demonstrated that TFF3 promotes ER+ MC cell migration and invasion [14], the functional and mechanistic aspects of whether TFF3 may contribute to metastasis of ER+ MC remain unclear.

In this study, we sought to determine if TFF3 is functionally associated with increased metastatic potential of ER+ MC cells. We first observed that TFF3 protein expressed in MC specimens was positively associated with metastasis and poor survival outcomes of patients with MC. Forced expression of TFF3 in ER+ MC cells was associated with increased invasion and metastatic seeding, as assessed *in vitro* and *in vivo*, and dependent on v-src avian sarcoma (Schmidt-Ruppin A-2) viral oncogene homologue-signal transducer and activator of transcription 3 (c-SRC- STAT3)-mediated repression of CDH1. Thus, functional inhibition of TFF3 may be considered as an adjuvant intervention to reduce metastatic seeding and ameliorate disease outcomes in patients with ER+ MC.

## Methods

### Cell culture and reagents

The human ER+ MC cell lines, MCF7 and T47D, were obtained from the American Type Culture Collection (ATCC, Rockville, MD, USA) and were cultured as per ATCC propagation instructions. Stable cell lines were freshly generated as previously described [14]. STAT3 activity inhibitor, JSI-124 and Stattic, was purchased from Sigma-Aldrich (Singapore). c-SRC family kinase inhibitor PP1, and the specific c-SRC kinase inhibitor PP2 was purchased from Sigma-Aldrich (Singapore); the structurally related non-inhibitory PP3 (50 μM) was purchased from Calbiochem (Singapore).

### Patient tissue microarrays and immunohistochemistry

The patient cohort used herein consists of 159 MC and 33 specimens of benign mammary disease (BMD) that underwent surgery at the First Affiliated Hospital of Anhui Medical University (AMU) (Hefei, Anhui, People's Republic of China) between 2001 and 2002 as previously described [19]. The Institutional Review Board of AMU approved the protocol for the use of patient specimens in this study [19]. Patient consent forms were obtained from all patients in accordance with the Declaration of Helsinki [19]. Immunohistochemistry (IHC) analysis was performed as previously described [19] using rabbit anti-pSTAT3 (Abcam, Cambridge, MA, USA) and rabbit anti-TFF3 polyclonal antibody [14]. The details of the cohort and IHC scoring methodology have previously been described in detail [19],[20] and in Additional file 1A. Survival analyses were performed on 126 patients in the cohort as 33 patients were lost to follow-up. The group of 126 patients used for survival analysis exhibited similar clinicopathologic features as the larger 159 patient cohort, with TFF3 positivity associated (*P* <0.05) with both increased tumour size and lymph node metastases.

### Plasmids and luciferase assay

Human *TFF3* expression and siRNA plasmid constructs have been previously described [14]. Human *STAT3* wild-type (WT), constitutively active variant (CA), dominant-negative variant (DN), and small interfering RNA (siRNA) plasmid constructs have been previously described [21]. The *α-2 macroglobulin* and *pGL2Basic-EcadK1* luciferase reporter construct was a generous gift from Dr. Xinmin Cao (Institute for Molecular and Cell Biology, Proteos, Singapore) and Dr Jean-Paul Thiery (Cancer Science Institute of Singapore, Singapore), respectively. Human *CDH1* expression vector was a generous gift from Dr A. Kraemer (UQ, Queensland, Australia). Luciferase assays were performed as previously described [22]. Briefly, transfections were carried out in triplicate using 1 μg of the appropriate luciferase reporter construct and empty vector per transfection along with 0.1 μg of *Renilla* luciferase construct as control for transfection efficiency. Luciferase activities were assayed after 24 hours of transfection using the Dual Luciferase Assay System (Promega Corp, Madison, WI, USA).

### PCR and quantitative-PCR

Total RNA was isolated from cells (cultured in 10% fetal bovine serum (FBS)) using TRIzol Plus RNA Purification system as previously described [23]. DNase I treatment, total RNA to complementary DNA, PCR, and qPCR assays was performed as previously described [23]. Gene expression analysis was performed as previously described [23] and the sequence of the primers are described in Additional file 1B. For the metastatic seeding studies, the following primers were used: *hHPRT* forward 5′-TTCCTTGGTCAGGCAGTATAATCC-3′ and reverse 5′-AGTCTGGCTTATATCCAACACTTCG-3′ *mgapdh* forward 5′-CTCACTCAAGATTGTCAGCAATG-3′ and reverse 5′-CACATTGGGGGTAGGAACAC-3′.

### Immunoblot and immunofluorescence

Immunoblot analysis was performed as previously described [23], using rabbit anti-TFF3 antibody [14]. Mouse anti-β-ACTIN, rabbit anti-p-c-SRC, mouse anti-c-SRC, and mouse anti-γ-CTNNG antibody was obtained from Santa Cruz Biotechnology, Santa Cruz, CA, USA. Mouse anti-CDH1, mouse anti-CDH2, rabbit anti-OCLN, mouse anti-VIM, mouse anti- ITGA6, rabbit anti-pSTAT3, and mouse anti-STAT3 antibody was obtained from Abcam, Cambridge, MA, USA. Cell extracts were resolved by SDS-PAGE and immunoblotted, with the respective antibodies, as previously described [23]. β-ACTIN was used as input control for cell lysate. The sizes of detected protein bands in kDa are shown on the left side.

Confocal laser scanning microscopy was performed as previously described [22]. Nuclei were visualised with VECTASHIELD mounting medium with DAPI (Vector Laboratories, Burlingame, CA, USA). Images were captured using a confocal microscope (Eclipse C1 Plus Confocal; Nikon Instrument Inc., Melville, NY, USA). Primary antibody, mouse anti-CDH1, and mouse anti-VIM was obtained from Abcam. Secondary antibody, Alexa Fluor 488 rabbit anti-mouse immunoglobulin (Ig)G and Alexa Fluor 568 rabbit anti-mouse IgG was obtained from Invitrogen, Singapore. Rhodamine-conjugated phalloidin (Sigma-Aldrich, St Louis, MO, USA) was used to visualize f-actin filaments.

### Oncogenicity assays

Cell migration and invasion assay were performed using BD BioCoat Matrigel invasion chambers (BD Biosciences, Bedford, MA, USA) according to the manufacturer's instructions and as previously described [23]. The colony scattering assay was performed as previously described [24]; at least one hundred colonies for each experimental condition were scored by phase contrast microscopy into three categories: compact (in which >90% of cells in a colony exhibit cell-cell contact), loose (in which 50 to 90% of cells exhibit cell-cell contact), and scattered (in which <50% of cells do so). Biological assays, an alamarBlue™ viability assay, anchorage-independent growth (soft agar colony formation and foci formation), two-dimensional and three-dimensional morphogenesis, and wound-healing assay was performed as previously described [23]. There was no significant change observed in MC cell invasion when cultured in media, exposed to dimethyl sulfoxide (DMSO), and/or transiently transfected with relative control plasmid (Additional file 2E). The collagen I adhesion assay was performed on a collagen I substrate-coated plate as per manufacturer's instructions (Invitrogen, Singapore). For the endothelial cell adhesion assay was performed as previously described [24], human microvascular endothelial cells (HMEC-1) cells were stained with CellTrace™ Calcein Red-Orange AM (50 μM) (Invitrogen), and MC cells stained with CellTrace™ Calcein Green AM (50 μM) (Invitrogen). Fluorescence measured by using a fluorescein filter set for CellTrace™ Calcein Green AM at Ex/Em = 504 nm/523 nm and CellTrace™ Red-Orange AM at Ex/Em = 577 nm/590 nm. Representative fluorescence images of each HMEC-1 (red) and MC cells (green) were documented. Endothelial transmigration assay was performed as previously described [24].

### Metastatic seeding assay

The *in vivo* work followed the animal care protocol USTCACUC1301013, which was approved by The Institutional Animal Care and Ethics Committee of The University of Science and Technology of China. Metastatic seeding assays were performed as previously described [24]. Balb/c nude mice (Shanghai Slaccas Co., Shanghai, China) (six weeks old) were used. 3 × 10^6^ viable MCF7-vector and MCF7-TFF3 cells (n = 6 for each group) were washed and harvested in phosphate-buffered saline (PBS) and subsequently injected into the lateral tail vein in a volume of 100 μl. After five weeks, mice were euthanized. Lung and liver tissue were resected for histological studies. Tissue samples were fixed in 4% paraformaldehyde, embedded in paraffin and 6 μM thick sections cut for haematoxylin and eosin (H&E) staining. For qPCR analysis, tissue samples were washed with PBS and frozen at -80°C in RNALater (Ambion, Austin, TX, USA) for RNA extraction.

### Statistics

Statistical analyses of patient's samples was performed using SPSS software (version 13.0; SPSS, Chicago, IL, USA), as previously described [19]. Briefly, differences between groups were compared using Pearson's chi-square test for qualitative variables and Student's *t* test for continuous variables. Kaplan-Meier curves were constructed to determine patient relapse-free survival (RFS) and overall survival (OS). Cox regression analyses were performed to evaluate differences of TFF3 expression factors in the risk of death. The statistical differences in survival among subgroups were compared using the log-rank test [25]. All numerical data are expressed as mean ± standard deviation (SD) from a representative experiment performed in triplicate. Statistical significance was assessed by using an unpaired two-tailed Student's *t* test (*P* <0.05 was considered as significant) by GraphPad Prism 5 (GraphPad Software, Inc, La Jolla, CA, USA).

## Results

### Higher expression of TFFprotein in MC is significantly associated with tumour size, lymph node metastasis, disease stage, and survival outcome of patients

**Figure 1:**
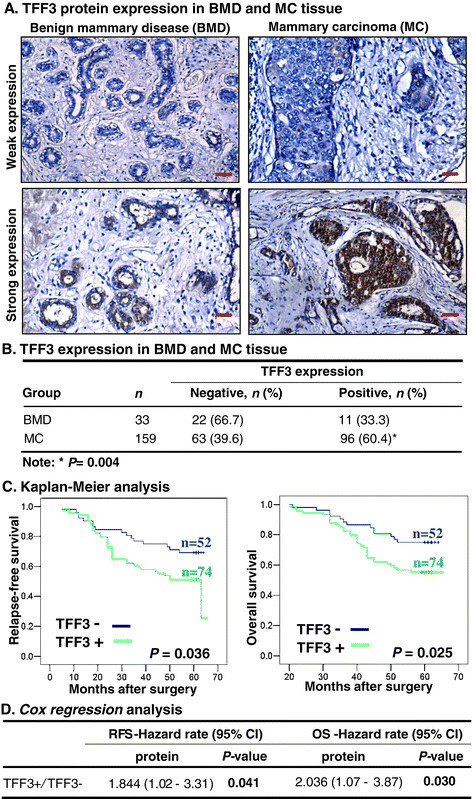
**Expression of TFF3 protein in MC. (A)** TFF3 expression was detected using immunohistochemistry (IHC) in benign mammary disease (BMD) and mammary carcinoma (MC) specimens. TFF3 was predominantly localised in the cytoplasm of carcinoma cells in MC tissue specimens. Representative images of TFF3 expression in estrogen receptor (ER) + MC tissue specimens were captured under X200 magnification. **(B)** Analysis of TFF3 expression in BMD and MC tissue specimens. Pearson's chi-square test was used to compare differences between groups. **(C)** Kaplan-Meier analysis of the significance of TFF3 expression on relapse-free survival (RFS) and overall survival (OS) of patients with MC. **(D)** Cox regression analysis was performed to evaluate the association of TFF3 expression with risk of death. The statistical differences among subgroups were compared using the log-rank test [25].

We first utilized IHC to determine the expression of TFF3 protein in tissue from BMD and MC. TFF3 expression was observed in 33.3% (n = 11/33) of BMD specimens. Weak or moderate expression of TFF3 protein was observed in the cytoplasm of epithelial cells of mammary ducts and acini. In contrast to BMD, 60.4% (n = 96/159) of MC specimens were positive for TFF3 protein expression, which represented a significantly higher percentage expression than that observed in BMD specimens (*P* = 0.004, Figure 1B). Moderate or strong expression of TFF3 was predominantly localised in the cytoplasm of carcinoma cells with an infrequently positive signal located in stromal (fibroblast and endothelial) cells (Figure 1A).

We next determined potential associations between TFF3 expression in MC and the clinicopathological characteristics of the tumour cohort. As observed in Table 1, expression of TFF3 protein was significantly associated with larger tumour size (*P* = 0.002), lymph node metastasis (*P* = 0.004), and higher disease stage (*P* = 0.040). No multicollinearity existed between variables examined. We further examined for a potential association of TFF3 expression with RFS or OS in the MC cohort using Kaplan-Meier survival analyses. We observed that patients with tumour expression of TFF3 exhibited significantly poorer RFS (*P* = 0.036) and OS (*P* = 0.025) compared to those patients whose tumours express no or low TFF3 (Figure 1C). Furthermore, Cox regression analyses demonstrated a significant association of tumour TFF3 expression with decreased RFS (*P* = 0.041) and OS (*P* = 0.030) (Figure 1D). Thus, TFF3 expression is frequently increased in MC, and associated with dissemination, as previously reported [11],[14] and predicts worse survival outcomes for patients with MC.

**Table 1:** **Associations between TFF3 expression in MC and the clinicopathological characteristics of the tumour cohort**

Parameter	***n***	TFF3 positive expression,***n***(%)	***P value***
Age (years)			
≤35	16	9 (56.3)	0.939
>35- ≤ 55	92	56 (60.9)	
>55	51	31 (60.8)	
Tumour size (cm)			
≤2	13	2 (15.4)	0.002
>2- ≤ 5	115	72 (62.6)	
>5	31	22 (71.0)	
Lymph node metastasis			
0	55	25 (45.5)	0.004
≤3	55	33 (60.0)	
>3	49	38 (77.6)	
Grade			
I	13	7 (53.8)	0.269
II	102	58 (56.9)	
III	44	31 (70.5)	
Stage			
I-II	85	45 (52.9)	0.040
III-IV	74	51 (68.9)	
ER^			
-	94	54 (57.4)	0.364
+	65	42 (64.6)	
PR^^			
-	90	54 (60.0)	0.912
+	69	42 (60.9)	
ERBB2			
low	107	69 (64.5)	0.129
high	52	27 (51.9)	

^ER + required at least 10% staining nuclei; ^^PR + required at least 10% staining nuclei. MC: mammary carcinoma; ER: estrogen receptor; PR: progesterone receptor; ERBB2: v-erb-b2 erythroblastic leukemia viral oncogene homologue 2.

### Forced expression of TFFin MC cells altered the mRNA levels of epithelial, mesenchymal, and metastatic-related gene markers

**Figure 2:**
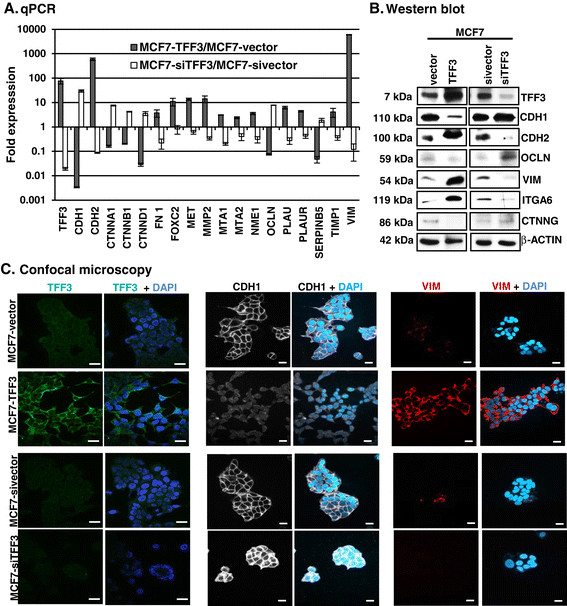
**Forced expression of TFF3 in MCF7 cells modulates the expression of epithelial, mesenchymal and metastatic-related gene markers. (A)** qPCR analyses of MCF7 cells with either forced or depleted expression of TFF3 for mRNA levels of key genes functionally involved in migration, invasion, metastasis, and epithelial-mesenchymal transition (EMT). Change in gene expression is expressed as fold difference, respectively. Fold change values are representative of three independent biological experiments. **(B)** Western blot analysis was used to assess the protein levels of epithelial and mesenchymal markers in MCF7 cells with either forced or depleted expression of TFF3 as described in Methods. **(C)** Confocal microscopic visualization of TFF3, CDH1, and VIM expression in MCF7 cells with either forced or depleted expression of TFF3. The green colour indicates TFF3 expression, white colour indicates CDH1 expression and red colour indicates the positive VIM expression; and the blue colour indicates nuclei. Images were captured under oil immersion X600 magnification. Statistical significance was assessed by using an unpaired two-tailed Student's *t* test (*P <0.05* was considered as significant) using GraphPad Prism 5. Columns are the mean of triplicate experiments; bars, ± SD.

Microarray datasets [26] available in the Oncomine database [27] demonstrated that the majority of MC cell lines were positive for *TFF3* mRNA expression. Among MC cell lines [26], 27/29 ER- MC and 18/18 ER+ MC cell lines were positive for *TFF3* mRNA expression (Additional file 2A). However, in general the expression of *TFF3* was higher in the ER+ cell line. We have previously demonstrated that TFF3 promotes MC cell invasion [14]. Herein, to determine the mechanism and functional role of TFF3 in metastatic progression of MC cells we newly generated ER+ cell lines, MCF7 (Figure 2) and T47D (Additional file 3), with either stable forced expression or siRNA-mediated depletion of TFF3. Significant differences in cell invasion were evident by the forced expression or siRNA-mediated depletion of TFF3 in both MCF7 and T47D cells (Additional file 2B,C,D, and E) and to a greater extent than previously reported [14].

For metastasis to occur, it has been proposed that cells lose epithelial characteristics and acquire a migratory mesenchymal phenotype with concomitant changes in gene expression [28]. Using qPCR analyses, we therefore quantified the mRNA levels of epithelial, mesenchymal, and metastatic-related genes in MC cells with either forced or depleted expression of TFF3. MCF7-TFF3 cells exhibited decreased mRNA levels of the epithelial gene markers, *CDH1*, *CTNNB1*, and *OCLN* relative to MCF7-vector cells. Concomitantly, MCF7-TFF3 cells exhibited increased mRNA levels of the mesenchymal gene markers, *CDH2*, *FN1*, *FOXC2*, and *VIM*, relative to MCF7-vector cells (Figure 2A). In contrast, MCF7-siTFF3 cells exhibited increased mRNA levels of the same epithelial gene markers and decreased mRNA levels of the same mesenchymal gene markers relative to MCF7-sivector cells (Figure 2A). Furthermore, we also quantified the mRNA levels of genes with a pivotal role in promoting cell invasion and metastasis of MC cells [29],[30]. MCF7-TFF3 cells exhibited increased mRNA levels of *MET*, *MMP2*, *MTA1, MTA2, NME1, PLAU, PLAUR*, *TIMP1*, and *PI5/SERPINB5* relative to MCF7-vector cells. Concordantly, MCF7-siTFF3 cells exhibited decreased mRNA levels of the same genes relative to MCF7-sivector cells (Figure 2A). Similar directional changes in the mRNA levels of the same epithelial, mesenchymal, and metastatic-related gene markers was observed also in T47D cells with either forced or depleted expression of TFF3 (Additional file 3A).

**Figure 3:**
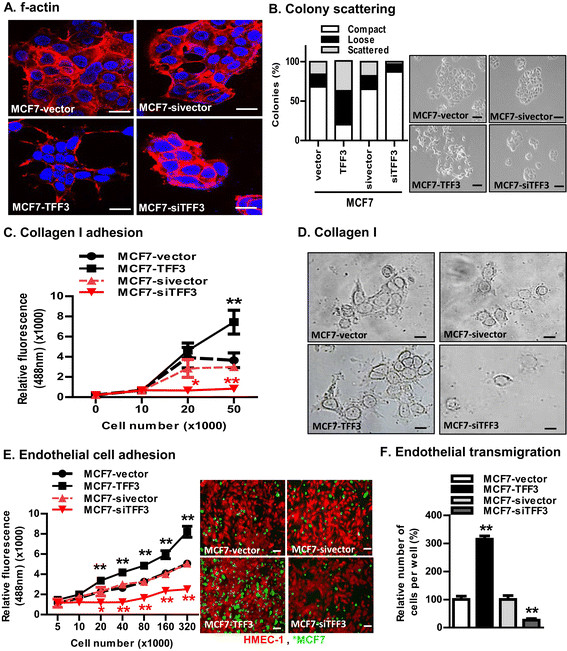
**Forced expression of TFF3 in MCF7 cells enhanced invasive phenotype. (A)** Confocal microscopic visualization of the f-actin arrangement in MCF7 cells with either forced or depleted expression of TFF3. The red colour indicates f-actin, and blue colour indicates nuclei stained with DAPI. Images were captured under oil immersion X600 magnification. **(B)** Distribution of compact, loose, and scattered colonies of MCF7 cells with either forced or depleted expression of TFF3 as described in Methods. Left side, representative images of compact, loose, and scattered monolayer adherent colonies of MCF7, as observed with either forced or depleted expression of TFF3 in MCF7 cells. **(C)** Capacity of MCF7 cells with either forced or depleted expression of TFF3 to adhere to a Collagen I matrix. **(D)** Morphology of MCF7 cells with either forced or depleted expression of TFF3 when cultured on a Collagen I matrix. **(E)**. Capacity of MCF7 cells with either forced or depleted expression of TFF3 to adhere to human microvascular endothelial cells (HMEC-1). The green colour indicates MCF7 cells (either forced or depleted expression of TFF3), and red colour indicates HMEC-1 cells. Images were captured under X100 magnification using a fluorescence microscope, as described in Methods. **(F)** Capacity of MCF7 cells with either forced or depleted expression of TFF3 to transmigrate through a HMEC-1 layer, as described in Methods. Statistical significance was assessed by using an unpaired two-tailed Student's *t* test (*P <0.05* was considered as significant) using GraphPad Prism 5. Columns are the mean of triplicate experiments; bars, ± SD. ^**^P <0.001, ^*^P <0.05.

Using western blot analysis, we further determined that MCF7-TFF3 cells exhibited decreased protein expression of CDH1, OCLN, and CTNNG; and higher protein levels of CDH2, VIM, and ITGA6 compared to MCF7-vector cells (Figure 3B). In contrast, MCF7-siTFF3 cells exhibited higher protein levels of CDH1, OCLN, and CTNNG; and lower protein levels of CDH2, VIM, and ITGA6 compared to MCF7-sivector (Figure 2B). T47D cells with either forced or depleted expression of TFF3 also exhibited similar changes in the protein levels of CDH1, CDH2, CTNNG, ITGA6, OCLN, and VIM (Additional file 3B). By immunofluorescence (IF), MCF7-TFF3 cells displayed prominent TFF3 expression in the cytoplasm of the cell compared to MCF7-vector cells (Figure 2C) and MCF7-siTFF3 cells displayed decreased TFF3 expression compared to MCF7-sivector cells. Furthermore, MCF7-TFF3 cells also displayed markedly reduced CDH1 expression and loss of the cell boundary localization of CDH1 compared to MCF7-vector cells (Figure 2C). In contrast, MCF7-siTFF3 cells displayed increased CDH1 expression compared to MCF7-sivector cells with prominent localization at the cell periphery (Figure 2C). MCF7-TFF3 cells displayed increased VIM expression compared to MCF7-vector cells, which displayed very low VIM expression (Figure 2C). VIM expression was not detected in MCF7-siTFF3 cells by IF. Immunofluorescent localization of CDH1 in T47D-TFF3 cells demonstrated reduced CDH1 expression and loss of cell boundary localization and concomitantly increased VIM expression when compared to their vector control cells (Additional file 3C and D). siRNA-mediated depletion of TFF3 in T47D cells increased expression of CDH1 and accentuated the cell boundary localization compared to their vector control cells (Additional file 3E). T47D-siTFF3 cells also exhibited decreased VIM expression compared to their vector control cells (Additional file 3D). Thus, TFF3 expression in ER+ MC cells resulted in decreased expression of epithelial and concomitantly, increased expression of mesenchymal and metastatic-related markers.

### Forced expression of TFFin MC cells enhanced invasive phenotype

We next visualised the subcellular distribution of filamentous (f-) actin using fluorescence microscopy. MCF7-TFF3 cells exhibited less stress fibres and enhanced accumulation of f-actin at the cell periphery coinciding with leading protrusions (Figure 3A). In contrast, the arrangement of stress fibres in MCF7-vector cells was condensed and localised to the edges of the cell membrane similar to the MCF7-sivector (Figure 3A) and parental MCF7 cells (data not shown). MCF7-siTFF3 cells did not show noticeable changes in clustering of f-actin compared to MCF7-sivector cells (Figure 3A). Similar but more pronounced alterations of the f-actin cytoskeleton and morphological changes were also observed in T47D cells with either forced or depleted expression of TFF3 (Additional file 4A).

In colony scattering assays by phase contrast microscopy, MCF7-TFF3 cells exhibited an elongated morphology with loss of cell-cell contact, with formation of multiple cellular protrusions (Figure 3B). In contrast, MCF7-vector cells exhibited epithelial characteristics and grew as defined groups of colonies with plentiful cell-to-cell contact similar to the MCF7-sivector (Figure 3B) and parental MCF7 cells (data not shown). MCF7-siTFF3 cells grew as noticeably small-size colonies with copious cell-to-cell contact (Figure 3B). MCF7-TFF3 cells formed 48% fewer compact colonies; and 27%, and 22% more loosely and scattered colonies, respectively, compared to MCF7-vector cells (Figure 3B). In contrast, MCF7-siTFF3 cells formed 22% more compact; and 7% and 17% fewer loose and scattered colonies, respectively, compared to MCF7-sivector cells (Figure 2B). In addition, T47D-TFF3 cells formed 44% fewer compact colonies; and 19% and 28% more loosely and scattered colonies, respectively, compared to T47D-vector cells. T47D-siTFF3 cells formed 7% more compact; and 3% and 10% fewer loose and scattered colonies, respectively, compared to T47D-sivector cells (Additional file 4B).

During the metastatic process, tumour cells adhere to, and invade through, the surrounding extracellular matrix and stroma [31],[32]. Collagen I is one of the main components of the mammary stromal matrix, to which the tumour cell adheres, and migrates through, to metastasize [32]. We therefore examined the effect of forced or depleted expression of TFF3 on the ability of MC cells to adhere to a Collagen I matrix. MCF7-TFF3 cells exhibited increased adhesion to a Collagen I matrix compared to MCF7-vector cells (Figure 3C). In contrast, MCF7-siTFF3 cells exhibited less adhesion to a Collagen I matrix compared to MCF7-sivector cells. We also examined the morphology of MC cells with either forced or depleted expression of TFF3 grown on a Collagen I matrix. When grown on a Collagen I matrix, MCF7-TFF3 cells exhibited a polarized morphology with formation of long protrusions compared to MCF7-vector cells, which displayed shorter and thicker cellular processes, similar to the MCF7-sivector cells (Figure 3D). MCF7-siTFF3 cells appeared mainly as single cells on a Collagen I matrix compared to MCF7-sivector cells, which grew as small groups. Similar directional changes in cell adhesion to a Collagen I matrix were observed in T47D cells with either forced or depleted expression of TFF3 (Additional file 4).

Culture on two-dimensional Matrigel is frequently utilised as one assay to determine the *in vitro* invasiveness of MC cells [33],[34]. We therefore examined the behaviour of MC cells with either forced or depleted expression of TFF3 when grown on two-dimensional Matrigel. Colonies formed by MCF7-TFF3 cells appeared much larger, flattened, and irregular in shape with cells extending from the colonies; compared to MCF7-vector cell-generated colonies, which grew as rounded small-size colonies, similar to the MCF7-sivector cells (Additional file 5A). In contrast, MCF7-siTFF3 cells appeared mainly as single cells on the Matrigel matrix compared to MCF7-sivector cell-generated colonies. The growth of MC cells on two-dimensional Matrigel was examined by use of the alamarBlue™ cell viability assay. MCF7-TFF3 cells exhibited increased cell viability when cultured on Matrigel-coated matrix compared to MCF7-vector cells. In contrast, MCF7-siTFF3 cells exhibited decreased cell viability compared to MCF7-sivector cells, when cultured on Matrigel-coated matrix (Additional file 5A). T47D cells with either forced or depleted expression of TFF3 also exhibited similar morphological changes when grown on two-dimensional Matrigel (Additional file 5), although the T47D-vector and T47D-sivector cells appeared to possess a more mesenchymal-like morphology compared to MCF7-vector or MCF7-sivector cells, respectively.

During the process of metastasis, carcinoma cells adhere to and migrate through endothelial cells that line the blood vessels in a process known as transendothelial migration [24],[35]. We therefore first determined the effects of TFF3 expression on MC cell heterotypic adhesion, a static cell-cell adhesion assay using HMEC-1 [24]. MCF7-TFF3 cells exhibited increased adhesion to the HMEC-1 cell monolayer compared to MCF7-vector cells (Figure 3E). In contrast, MCF7-siTFF3 cells exhibited reduced cell adhesion to the HMEC-1 cell monolayer compared to MCF7-sivector cells. We next examined the effect of forced or depleted expression of TFF3 in MC cells on transendothelial migration through the HMEC-1 cell monolayer. MCF7-TFF3 cells exhibited increased transendothelial migration through the HMEC-1 cell monolayer compared to MCF7-vector cells (Figure 3F). In contrast, MCF7-siTFF3 cells displayed reduced transendothelial migration through the HMEC-1 cell monolayer compared to MCF7-sivector cells (Figure 3F). T47D cells with forced expression of TFF3 exhibited increased adhesion to, and transendothelial migration through the HMEC-1 monolayer compared to their vector control cells (Additional file 5). siRNA-mediated depletion of TFF3in T47D cells decreased adhesion to, and transendothelial migration through, the HMEC-1 monolayer compared to their vector control cells (Additional file 5). Thus, TFF3 expression stimulated MC cell adhesion to extracellular matrices and endothelial cells, and transmigration through an endothelial cell barrier.

### Forced expression of TFF3 in MCF7 cells stimulates metastatic seeding

**Figure 4:**
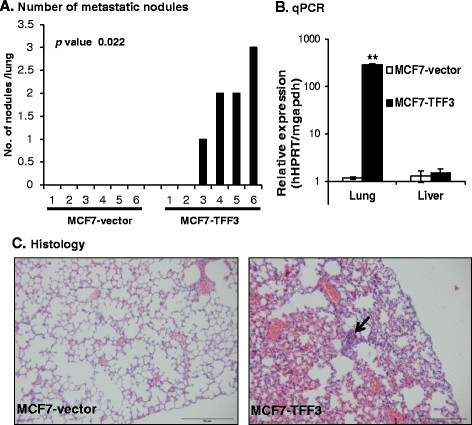
**Forced expression of TFF3 in MCF7 cells stimulates metastatic seeding. (A)** Metastatic nodules per lung of BALB/c nude mice (n = 6 each group) that received lateral tail vein injection of either MCF7-vector or MCF7-TFF3 cells. **(B)** Total RNA was isolated from lung and liver tissue of individual mice that received lateral tail vein injection of either MCF7-vector or MCF7-TFF3 cells. qPCR was performed to measure the mRNA expression of human hypoxanthine-guanine phosphoribosyltransferase (*hHPRT*) in the lung and liver of mice. Mouse glyceraldehyde 3-phosphate dehydrogenase (*mgapdh*) was used as an internal control. The relative expression of *hHPRT* vs *mgapdh* was calculated. **(C)** Illustrative haematoxylin and eosin (H&E)-stained sections of lungs from mice that received lateral tail vein injection of MCF7-vector or MCF7-TFF3 cells. Arrow indicates metastatic nodules. Statistical significance was assessed by using an unpaired two-tailed Student's *t* test (*P <0.05* was considered as significant) using GraphPad Prism 5. Columns are the mean of triplicate experiments; bars, ± SD. ^**^P <0.001, ^*^P <0.05.

To determine whether TFF3 contributes to the metastatic seeding of MC cells, MCF7-vector and MCF-TFF3 cells were injected into the tail vein of BALB/c nude mice (n = 6 each group) and examined their ability to form metastatic nodules (Figure 4A). Histological analyses determined that 4/6 mice injected with MCF7-TFF3 cells formed metastatic nodules in the lungs (Figure 4C). Moreover, MCF7-TFF3 cells gave rise to ≥1 nodule per lung per mice, whereas no metastatic nodules were detected in the lungs of mice injected with MCF7-vector cells. No metastatic nodules were detected in the liver of mice injected with either MCF7-vector or MCF7-TFF3 cells.

We also extracted RNA from lung and liver of mice injected with MCF7-vector or MCF7-TFF3 cells and performed qPCR to quantitate the relative expression of human hypoxanthine-guanine phosphoribosyltransferase (*hHPRT*) mRNA in both organs. We observed that the level of *hHPRT* mRNA expression was increased >100-fold in the lungs of animals injected with MCF7-TFF3 cells compared with MCF7-vector cells. Minimal *hHPRT* mRNA was detected in the liver of mice injected with MCF-vector cells and no significant differences of *hHPRT* mRNA expression were observed in liver tissue from mice injected with either MCF7-vector or MCF7-TFF3 cells. Mouse glyceraldehyde 3-phosphate dehydrogenase (*mgapdh*) was used as an internal control (Figure 4B). Thus, forced expression of TFF3 in MCF7 cells enhanced the capacity for MC cell survival in the circulation, extravasation, and colonization of the targeted host organ (Lung).

### Repression of CDH1 expression is essential for TFF3-stimulated invasion of MC cells

Loss of CDH1 expression, or CDH1 dysfunction, is associated with the loss of cell-cell interaction stimulating an invasive cell phenotype and metastasis [28],[31],[36]. We have previously reported that TFF1 repressed CDH1 expression in prostate carcinoma cells to promote cell invasion [37],[38]. To determine if decreased CDH1 expression modulated TFF3-stimulated MC cell invasion, we therefore assessed the invasive capacity of MCF7-vector/MCF7-TFF3 and MCF7-sivector/MCF7-siTFF3 cells with forced expression of CDH1. As previously described [14], MCF7-TFF3 cells exhibited significantly increased invasion through Matrigel compared to MCF7-vector cells. Forced expression of CDH1 in MCF7 cells reduced both basal and the TFF3-stimulated invasion through Matrigel (Additional file 6A). Combined, forced expression of CDH1 and depleted expression of TFF3 in MCF7 cells exhibited further decreased capacity of invasion through Matrigel. Forced expression of CDH1 in T47D-TFF3 cells similarly abrogated TFF3-stimulated cell invasion (Additional file 6A). Thus, reduction of CDH1 expression in ER+ MC cells is required for TFF3-stimulated cell invasion.

### Forced expression of TFF3 in MC cells increased phosphorylation of STAT3 to promote invasion

**Figure 5:**
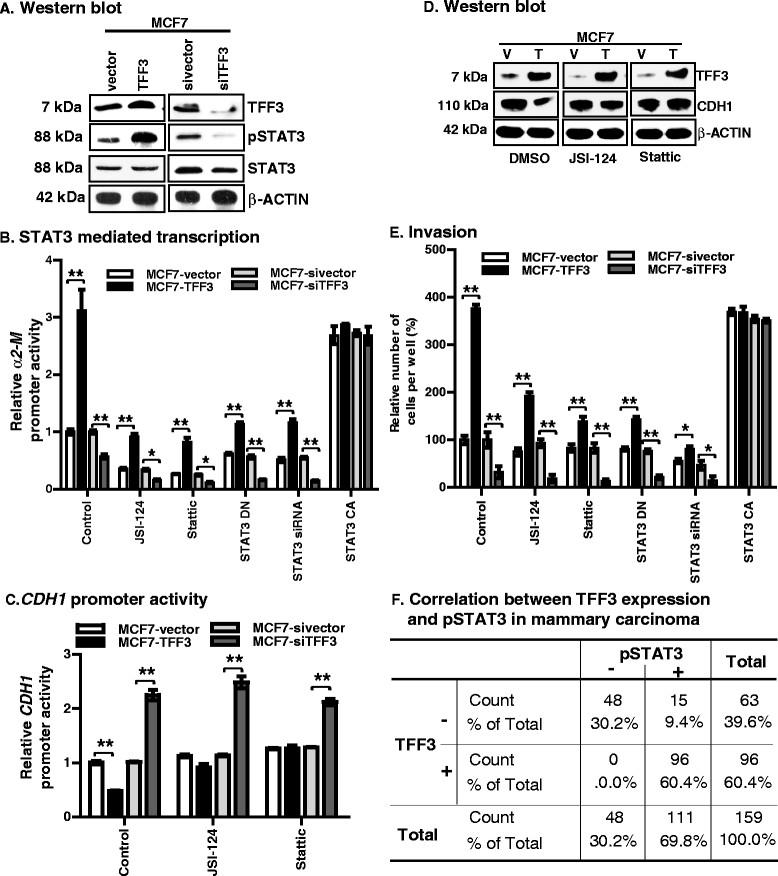
**Forced expression of TFF3 in MCF7 cells increased phosphorylation of STAT3 to promote invasion; and TFF3 expression significantly correlates with pSTAT3 expression in MC patients' tissue. (A)** Western blot analysis was used to assess the levels of pSTAT3 and STAT3 in MCF7 cells with either forced or depleted expression of TFF3 as described in Methods. **(B)** STAT3 mediated transcription, *α2-M* promoter activity in MCF7 cells with either forced or depleted expression of TFF3, on exposure to JSI-124 (0.2 μM) or Stattic (2 μM) inhibitor; and transiently transfected with *STAT3 DN*, *STAT3-siRNA* or *STAT3 CA*. The luciferase assay was performed as described in Methods. **(C)** *CDH1* promoter activity in MCF7 cells with either forced or depleted expression of TFF3, on exposure to JSI-124 (0.2 μM) or Stattic (2 μM) inhibitor. The luciferase assay was performed as described in Methods. **(D)** Western blot analysis was used to assess the levels of CDH1 in MCF7 cells with forced expression of TFF3 on exposure to JSI-124 (0.2 μM) or Stattic (2 μM) inhibitor as described in Methods. **(E)** Invasive capacity of MCF7 cells with either forced or depleted expression of TFF3, on exposure to JSI-124 (0.2 μM) or Stattic (2 μM); and transiently transfected *STAT3 DN*, *STAT3-siRNA* or *STAT3 CA*. Cell invasion was evaluated using a Transwell assay. **(F)** Correlation between TFF3 expression and pSTAT3 in the MC cohort utilized in Figure 1B. Pearson's chi-square test was used to compare the differences between groups. Statistical significance was assessed by using an unpaired two-tailed Student's *t* test (*P <0.05* was considered as significant) using GraphPad Prism 5. Columns are mean of triplicate experiments; bars, ± SD. ^**^P <0.001, ^*^P <0.05.

TFF3 has been previously reported to increase phosphorylation (Tyr^705^) of STAT3 in colon carcinoma cell lines [39]. Moreover, published reports have also observed that increased STAT3 activity represses CDH1 expression in MC cells [40],[41]. Using western blot analysis, we first evaluated the level of phosphorylated (p) STAT3 (at Y^705^) in MCF7 cells with either forced or depleted expression of TFF3. MCF7-TFF3 cells exhibited increased levels of pSTAT3 compared to MCF7-vector cells (Figure 5A). In contrast, MCF7-siTFF3 cells exhibited reduced levels of pSTAT3 compared to MCF7-sivector cells. The protein levels of total STAT3 were not significantly altered in MCF7 cells with either forced or depleted expression of TFF3 when compared with their vector control cells. Similar directional changes were observed with pSTAT3 levels in T47D cells with either forced or depleted expression of TFF3 (Additional file 7A).

We subsequently assessed STAT3 mediated transcriptional activity using an *α2-macroglubulin* (α*2-M*) promoter in MCF7 cells with either forced or depleted expression of TFF3. The α2*-M* reporter construct contains a fragment of the α*2-M* gene promoter (-215 to +8 bp) to which STAT3α binds and induces transcription of this gene [42]. MCF7-TFF3 cells exhibited increased *α2-M* promoter activity when compared to MCF7-vector cells. On exposure to the STAT3 activity inhibitor, JSI-124 or Stattic, both MCF7-vector and MCF7-TFF3 cells exhibited decreased basal and TFF3-stimulated *α2-M* promoter activity (Figure 5B). Moreover, *STAT3* DN or *STAT3-siRNA* constructs were used to assess whether the TFF3-mediated increase in *α2-M* promoter activity was STAT3 dependent. The *STAT3 DN* construct possesses an amino acid substitution at Tyr^705^ essential for STAT3 activity and this mutation renders STAT3 inactive [43]. TFF3 stimulated increases in *α2-M* promoter activity were largely inhibited by transient-transfection of either *STAT3 DN* or *STAT3-siRNA* in MCF7 cells with forced expression of TFF3 (Figure 5B). Hence, inhibition of STAT3 or depletion of STAT3 in MCF7 cells attenuated TFF3-stimulated *α2-M* promoter activity compared to their vector control cells (Figure 5B). In contrast, transient-transfection of a CA *STAT3* construct in MCF7-vector or MCF7-TFF3 cells equivalently increased levels of *α2-M* promoter activity. Moreover, transient-transfection of *STAT3 CA* in MCF7 cells prevented the loss of *α2-M* promoter activity in cells with siRNA-mediated depletion of TFF3 (Figure 5B). Forced expression of TFF3 in T47D cells similarly increased *α2-M* promoter activity whereas siRNA-mediated depletion of TFF3 decreased *α2-M* promoter activity (Additional file 7B).

To determine the association between increased STAT3 activity and CDH1 repression by TFF3, we evaluated the promoter activity of *CDH1* in MC cells. MC cells with either forced or depleted expression of TFF3 were transiently transfected with a reporter plasmid containing the luciferase gene under the control of a 233 bp fragment of the *human CDH1* promoter (sequences from -108 to +125). MCF7-TFF3 cells exhibited repressed *CDH1* promoter activity compared to MCF7-vector cells. On exposure to JSI-124 or Stattic, the TFF3-mediated repression of *CDH1* promoter activity was largely prevented in MCF7-TFF3 cells (Figure 5C). MCF7-siTFF3 cells exhibited increased *CDH1* promoter activity compared to their vector control cells. No further enhancement of *CDH1* promoter activity was observed in either MCF7-vector or MCF7-sivector cells after exposure to JSI-124 or Stattic. As above, we have demonstrated that forced expression of TFF3 in MC cells resulted in decreased expression of *CDH1* mRNA compared to their vector control cells. We therefore evaluated CDH1 protein levels in MCF7-vector and MCF7-TFF3 cells after exposure to JSI-124 or Stattic. On exposure to JSI-124 or Stattic, MCF7-TFF3 cells exhibited increased CDH1 protein levels relative to vehicle (DMSO)-exposed cells (Figure 5D). Hence, TFF3-mediated repression of CDH1 protein expression was abrogated after exposure to STAT3 inhibitors. No significant change in CDH1 protein levels in MCF7-vector or MCF7-sivector cells was observed after exposure to JSI-124 or Stattic compared to vehicle (DMSO)-exposed cells. Forced expression of TFF3 in T47D cells also decreased CDH1 promoter activity and siRNA-mediated depletion increased CDH1 promoter activity. Exposure of T47D-TFF3 cells to JSI-124 or Stattic repressed both CDH1 promoter activity and protein expression (Additional file 7C and D).

By use of confocal laser scanning microscopy (Figure 2C) we observed that forced expression of TFF3 in MCF7 cells reduced CDH1 expression and resulted in loss of cell boundary localization. We therefore also assessed the expression and localization of CDH1 in MCF7 cells after exposure to JSI-124 or Stattic. Upon exposure to JSI-124 or Stattic, both MCF7-TFF3 and MFC7-vector cells exhibited increased expression of CDH1 and accentuated cell boundary localisation compared to their vehicle control cells (Additional file 3D). Specifically, exposure of MCF7-TFF3 cells to either JSI-124 or Stattic enhanced CDH1 expression and restored the localisation of CDH1 to the cell boundary. Exposure of T47D-TFF3 cells to JSI-124 or Stattic also increased CDH1 expression and restored the cell boundary localisation of CDH1, although not as prominently as that observed in MCF7-TFF3 cells (Additional file 3D).

We next determined the effect of STAT3 inhibition on the invasion of MCF7 cells with either forced or depleted expression of TFF3. As described above, MCF7-TFF3 cells exhibited increased invasion through Matrigel when compared to MCF7-vector cells. Inhibition of STAT3 activity, using JSI-124 or Stattic, decreased both basal and TFF3-stimulated invasion in MCF7-vector and MCF7-TFF3 cells (Figure 5D). Moreover, the stimulatory effect of TFF3 on cell invasion was largely abrogated by transient-transfection of either *STAT3 DN* or *STAT3-siRNA* in MCF7-TFF3 cells (Figure 5E). In contrast, transient-transfection of *STAT3 CA* in MCF7-vector cells enhanced their basal capacity for cell invasion equally to MCF7-TFF3 cells. Transient-transfection of *STAT3 CA* in MCF7-siTFF3 cells completely abrogated the effect of TFF3 depletion on cell invasion. Hence, depletion or inhibition of STAT3 activity in MCF7 cells attenuated TFF3-stimulated cell invasion through Matrigel (Figure 5E). Depletion or inhibition of STAT3 activity in T47D cells with forced expression of TFF3 also largely abrogated the ability of STAT3 to stimulate MC cell invasion (Additional file 7E).

In addition, we also examined for a potential association of TFF3 and pSTAT3 protein expression in the cohort of MC patients used above. IHC analysis showed that both TFF3 and pSTAT3 protein were highly expressed in MC tissue specimens with 60.4% positive for TFF3 (as Figure 1B) and 69.8% positive for pSTAT3 (Figure 5F). Of those tumours negative for TFF3 expression, 23.8% were positive for pSTAT3. In contrast, all tumours positive for TFF3 expression were also positive for pSTAT3. Furthermore, we compared the relationship between TFF3 expression and pSTAT3 in MC tissue samples using Pearson correlation and observed a Pearson correlation of 0.812 (*P* value <0.01) (Figure 5F). Thus, TFF3 expression in ER+ MC cells promotes an invasive phenotype through increased STAT3 phosphorylation and transcriptional repression of CDH1.

### Forced expression of TFF3 in MC cells enhanced phosphorylation of c-SRC that subsequently increased STAT3 activity to promote invasion

**Figure 6:**
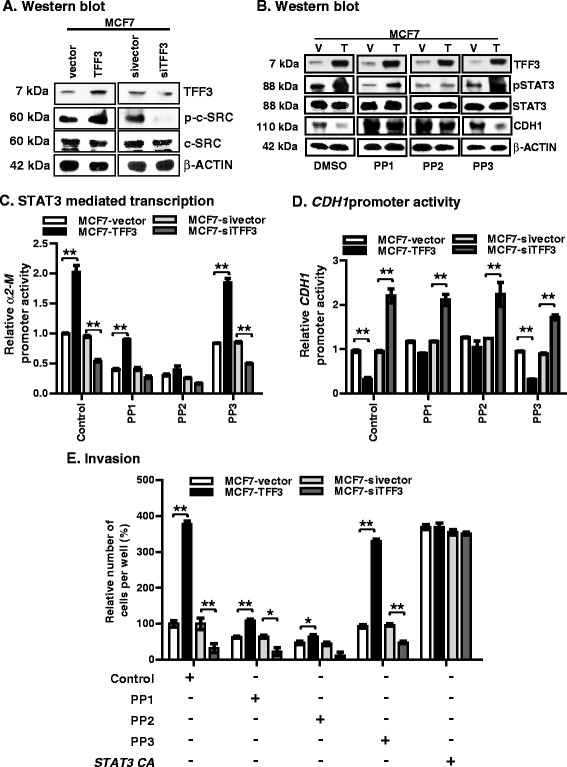
**Forced expression of TFF3 in MCF7 cells enhanced phosphorylation of c-SRC that subsequently increased STAT3 activity to promote invasion. (A)** Western blot analysis was used to assess the levels of p-c-SRC and c-SRC in MCF7 cells with either forced or depleted expression of TFF3 as described in Methods. **(B)** Western blot analysis was used to assess the levels of pSTAT3, STAT3, and CDH1 in MCF7 cells with forced expression of TFF3 on exposure to PP1 (5 μM), PP2 (2 μM) or PP3 (50 μM) as described in Methods. **(C)** *CDH1* promoter activity in MCF7 cells with either forced or depleted expression of TFF3, on exposure to PP1 (5 μM), PP2 (2 μM) or PP3 (50 μM). The luciferase assay was performed as described in Methods. **(D)** *α2-M* promoter activity in MCF7 cells with either forced or depleted expression of TFF3, on exposure to PP1 (5 μM), PP2 (2 μM), or PP3 (50 μM). The luciferase assay was performed as described in Methods. **(E)**. Invasive capacity of MCF7 cells with either forced or depleted expression of TFF3, on exposure to PP1 (5 μM), PP2 (2 μM) or PP3 (50 μM); and/or in combination with transiently transfected *STAT3 CA*. Cell invasion was evaluated using a Transwell assay. Statistical significance was assessed by using an unpaired two-tailed Student's *t* test (*P <0.05* was considered as significant) using GraphPad Prism 5. Columns are the mean of triplicate experiments; bars, ± SD. ^**^P <0.001, ^*^P <0.05.

c-SRC has been reported to increase STAT3 activity in carcinoma cells including MC [44],[45]. Therefore, to determine if TFF3 utilized c-SRC to stimulate tyrosine phosphorylation of STAT3, we evaluated p-c-SRC (at Y^416^) levels in MCF7 cells with either forced or depleted expression of TFF3 using western blot analysis. MCF7-TFF3 cells exhibited increased levels of p-c-SRC compared to MCF7-vector cells (Figure 6A). In contrast, MCF7-siTFF3 cells exhibited decreased levels of p-c-SRC levels compared to MCF7-sivector cells. The protein levels of total c-SRC were not consistently altered in MCF7 cells with either forced or depleted expression of TFF3 when compared to their vector control cells. TFF3 also promotes phosphorylation of c-SRC in T47D cells (Additional file 8).

Furthermore, we subsequently evaluated pSTAT3 protein levels in MCF7-vector and MCF7-TFF3 cells after exposure to the c-SRC family kinase inhibitor, PP1 or c-SRC inhibitor, PP2. On exposure to PP1 or PP2, both MCF7-vector and MCF7-TFF3 cells exhibited decreased basal and TFF3-stimulated pSTAT3 levels relative to vehicle (DMSO) and/or PP3 (the inactive structural homologue)-exposed cells (Figure 6B). Relatively, PP2 exposure prevented TFF3-stimulated pSTAT3 levels more effectively in MCF7-TFF3 cells compared to PP1. Protein levels of total STAT3 were not significantly altered on exposure to PP1, PP2, and/or PP3 in either MCF7-TFF3 or MCF7-vector cells. Also, on exposure to PP1 or PP2, both T47D-vector and T47D-TFF3 cells exhibited decreased basal and TFF3-stimulated pSTAT3 levels relative to vehicle (DMSO)-exposed cells (Figure 6B). Again, PP2 exposure also prevented TFF3-stimulated pSTAT3 levels more effectively in T47D-TFF3 cells than PP1-exposed cells. There was some diminution of basal and TFF3-stimulated pSTAT3 levels upon exposure of T47D-vector and T47D-TFF3 cells to PP3, although a prominent activation of STAT3 by TFF3 was maintained. Protein levels of total STAT3 were not consistently altered in either T47D-TFF3 or T47D-vector cells on exposure to PP1, PP2, or PP3. In addition, on exposure to either PP1 or PP2, both MCF7-vector and MCF7-TFF3 cells exhibited increased CDH1 protein levels relative to vehicle (DMSO)- or PP3-exposed cells (Figure 6B). Hence, TFF3-mediated repression of CDH1 protein expression in MCF7 cells was prevented after exposure to PP1 or PP2. Exposure of T47D-vector cells to PP1 or PP2 did not increase CDH1 expression but did prevent expression of CDH1 expression in T47D-TFF3 cells (Additional file 8B).

We next evaluated *α2-M* and *CDH1* promoter activity in MCF7 cells with either forced or depleted expression of TFF3 after exposure to PP1, PP2, or PP3. There was no significant effect on TFF3-stimulated *α2-M* promoter activity in MCF-7 cells upon exposure to PP3. Both MCF7-vector and MCF7-TFF3 cells exhibited decreased basal and TFF3-stimulated *α2-M* promoter activity when exposed to PP1 or PP2 compared to vehicle (DMSO)- or PP3-exposed cells (Figure 6C). PP2 exposure prevented TFF3-stimulated *α2-M* promoter activity more effectively in MCF7-TFF3 cells compared to PP1. Combined, inhibition of c-SRC and depleted expression of TFF3 in MCF7 cells further attenuated the *α2-M* promoter activity compared to their vector control cells. T47D cells with either forced or depleted expression of TFF3 predominantly exhibited similar directional changes in *α2-M* promoter activity on exposure to PP1 or PP2 (Additional file 8C).

Upon exposure to PP1 or PP2, the TFF3-mediated repression of *CDH1* promoter activity was largely prevented in MCF7-TFF3 cells (Figure 6D) and PP3 was without effect. MCF7-siTFF3 cells exhibited increased *CDH1* promoter activity compared to vector control cells and which was not consistently affected by exposure to PP1, PP2, or PP3. A marginal increase in *CDH1* promoter activity was observed in MCF7-vector or MCF7-sivector cells after exposure to PP1 or PP2. Similarly, TFF3-mediated repression of the CDH1 promoter in T47D cells was abrogated upon exposure to PP1 or PP2 but not PP3 (Additional file 8D).

We next assessed the invasive capacity of MC cells with either forced or depleted expression of TFF3 upon exposure to PP1, PP2, or PP3. Exposure to PP3 marginally decreased the TFF3-stimulated cellular invasion in MCF7 cells compared to vehicle (DMSO)-exposed cells. On exposure to PP1 or PP2, MCF7-vector and MCF7-TFF3 cells exhibited decreased basal and TFF3-stimulated invasion compared to vehicle (DMSO)-or PP3-exposed cells. Relatively, the inhibitory effect was greater for PP2 compared to PP1 (Figure 6E). Inhibition of c-SRC in MCF7 cells with depletion of TFF3 in MCF7 cells further attenuated invasion when compared to vehicle (DMSO)- or PP3-exposed cells (Figure 6E). T47D cells with either forced or depleted expression of TFF3 exhibit similar directional changes in cellular invasion through Matrigel on exposure to PP1 or PP2 (Additional file 8E). Thus, forced expression of TFF3 in ER+ MC cells promotes an invasive phenotype through increased c-SRC activation of STAT3 leading to downregulation of CDH1 expression.

## Discussion

Herein, we have observed that TFF3 expression is frequently increased in MC and its higher expression predicts metastasis and poor survival outcome of patients. Forced expression of TFF3 in ER+ MC cells resulted in the acquisition of a mesenchymal phenotype with subsequent enhanced migratory and invasive behaviour. *In vivo*, forced expression of TFF3 in MCF7 cells stimulated metastatic nodule formation in the lungs of animals. Mechanistically, TFF3 expression in ER+ MC cells stimulated phosphorylation of c-SRC, which subsequently increased STAT3 activity and led to the downregulation of CDH1 to promote cell invasion. Together, these results are consistent with previously published reports that observed correlations between TFF3 expression and local or metastatic dissemination of MC cells [11],[15]-[17]. However, we have further defined survival outcomes associated with TFF3 expression, demonstrated a functional involvement of TFF3 in metastasis of ER+ MC cells and defined the mechanisms utilized. Hence, TFF3 expression in ER+ MC promotes metastasis.

Whilst a number of studies have reported an association of TFF3 expression with micrometastasis/metastasis of MC [11],[15]-[17], there exists a paucity of studies on the association of TFF3 expression with survival outcome for patients with MC. Higher *TFF3* mRNA levels have previously been reported to predict a worse survival outcome of patients with ER+ MC treated with tamoxifen [14]. We have further observed herein that high levels of TFF3 protein correlated with a poorer RFS and OS of patients with MC both by Cox regression analyses. Such observations are consistent with recent reports in gastric carcinoma where TFF3 expression was reported to predict poor survival [46],[47] and was associated with vascular invasion and lymph node metastasis [47]. Similarly, we herein and others [11],[15],[17] have also reported that TFF3 expression in the primary tumour predicted lymph node metastasis of MC. Moreover, it was reported that TFF3 expression in tumour cells disseminated to lymph nodes was significantly higher than that observed in the primary tumour [11],[18] and further significantly enhanced in tumour cells that penetrated the node capsule. In contrast, a recent histopathological study in type 1 endometrial carcinoma observed that tumour expression of TFF3 predicted lack of lymphovascular invasion, a lower recurrence and higher survival of patients [48]. It is relevant to note, however, that endometrial adenocarcinoma is an estrogen-dependent tumour and hence similar to MC [49], ER positivity is associated with a favourable prognosis [48]. It was noted that expression of TFF3 in endometrial adenocarcinoma was positively correlated with ER expression in the studied cohort [48]. In the MC cohort studied herein, we did not observe a significant association of TFF3 with ER status nor did we observe any significant differences in survival between the ER+ or ER- subgroups (data not shown). It is plausible that in other cohorts of MC where TFF3 expression is more tightly associated with ER positivity that the expression of TFF3 may be associated with a favourable prognosis, as are other estrogen-regulated genes such as BCL-2 [50],[51] and TFF1 [52]. Hence, it is important to note that BCL-2 is oncogenic and BCL-2 inhibitors experimentally reduce tumour growth [53], yet BCL-2 expression is associated with a favourable prognosis in MC due to its association with the ER+ subtype. In any case, TFF3 is prominently expressed in the molecular apocrine subtypes of ER- MC [12],[13]. It should be noted that our cohort is entirely Han Chinese in origin (compared to those based on Caucasian populations [11]), the majority of patients are aged below 55 years (68%) and the cohort contains a predominance of ER- (59%) and progesterone receptor negative (PR-) (56%) tumours. Among ER- and PR- tumours, 58% (44 out of 75 cases) tumours are TFF3 positive. Molecular apocrine tumours are ER-PR- tumours characterised by the continued expression of estrogen responsive genes [54] and constitute 12 to 37% of ER- tumours. Regardless of the discrepancies in the histopathological analyses and between tumours of different organ origin, we have demonstrated that TFF3 is a functional promoter of metastatic dissemination of MC cells.

Herein, we have observed that TFF3-stimulated invasion of MC cells was promoted through c-SRC-STAT3-mediated repression of CDH1. Constitutive activation of STAT3 has been observed in a wide range of human solid carcinoma including MC, and is commonly associated with worse prognosis [55]-[57]. Persistently activated STAT3 has been reported to modulate the transcription of a number of target genes involved in metastatic progression of MC, such as TWIST1, SNAIL, TENASCIN-C, IL-8, and including CDH1 [55],[56],[58]-[60]. TGF*β* and/or EGF/EGFR-mediated STAT3 activation also has been observed to possess a pivotal role in EMT through increased TWIST1 expression in MC cells [57],[61]. TWIST2 another member of the TWIST family proteins and forced expression of TWIST2 in MCF10A cells resulted in increased STAT3 activity and downregulation of CDH1 that subsequently promoted EMT and enhanced the self-renewal of MC stem-like cells [40]. Concordant with these reports, we have also observed increased expression of TWIST1 protein in MC cells consequent to forced expression of TFF3 (unpublished data). MC stem-like cells have been postulated to play a pivotal role in acquired resistance to chemotherapy and to recurrence of disease [62]. Indeed, acquired resistance to endocrine therapy or chemotherapy is recognised as one hallmark characteristic of MC stem-like cells [62]. Such a notion is concordant with our previous report demonstrating the functional relevance of elevated levels of TFF3 protein in acquired tamoxifen-resistant MCF7 cells [14]. Herein, we demonstrated that high levels of TFF3 protein in ER+ MC cells increased STAT3 activity. Side populations with cancer stem-like cells (CSC)-like activity from MCF7 cells exhibit high STAT3 activity, which is required for maintenance of the CSC population and/or behaviour [63]. Enhanced STAT3 activity in the CD44^high^ CD24^low/negative^ subpopulation from MCF7 cells has been reported to promote intrinsic resistance to tamoxifen [64]. MCF7 cells with acquired tamoxifen resistance have also been demonstrated to possess increased STAT3 activity, which promotes cell survival as one mechanism to generate resistance to tamoxifen [65]. An increased level of TFF3 protein in MCF7 cells with acquired docetaxel resistance has also been observed (unpublished preliminary data). Moreover, siRNA-mediated depletion of TFF3 in MCF7 cells enhanced sensitivity to docetaxel compared to their vector control cells (unpublished preliminary data). Such observations are concordant with previous reports describing a positive correlation between STAT3 activity and chemotherapeutic resistance through increased expression of the anti-apoptotic protein BCL-2 in metastatic MC cells [66]. Concordantly, we have also observed that forced expression of TFF3 in MCF7 cells resulted in increased mRNA levels of *BCL2* and BCL2 protein [14]. Additionally, STAT3 activity is enhanced in paclitaxel-resistant ovarian carcinoma cells to maintain the resistant state of the cells [67]. Our current findings therefore rationally raise the possibility that TFF3 may potentially contribute to the self-renewal of MC stem-like cells.

The EMT process associated with phenotypic and molecular alterations that are positively correlated with increased metastatic capacity of carcinoma cells and the MC stem-like cell phenotype [62]. Concordant with published characteristics of EMT [28], we have herein observed that forced expression of TFF3 in ER+ MC cells decreased expression of CDH1, OCLN, and CTNNG; and concomitantly increased expression of VIM, and CDH2. Previous reports have also suggested that the motogenic effects of TFF1 and TFF3 may be mediated through decreased expression of CDH1 [38],[68],[69]. A number of studies have also demonstrated decreased expression of CDH1 in response to TFF1 [37],[38] or TFF3 [68],[70] in other cell systems. Decreased expression of CTNNG and OCLN in ER+ MC cells results in increased invasion and *in vivo* dissemination of MC cells [71],[72]. Furthermore, loss of CDH1 is often accompanied by the increased expression of CDH2 in MC [73]. Functionally, homophilic interactions between CDH2 expressed in MC cells and vascular endothelial cells have been reported to facilitate MC cell intravasation and extravasation; and to promote metastatic dissemination of MC cells [73]. We have observed herein that TFF3 promotes both vascular endothelial attachment and endothelial transmigration of MC cells and the observed TFF3-stimulated increase in CDH2 may be one mechanism for this. Mechanistically, the STAT3-regulated transcription factors [60], SNAIL and TWIST1 had been reported to coordinately regulate the expression of CDH1 and CDH2 [28]. An increased expression of SNAIL in MC is positively associated with poor RFS and recurrence [74]. Moreover, studies on MC patient cohorts have also supported a link between increased expression of SNAIL, TWIST1, and reduced RFS of ER+ MC patients [75]. In a large retrospective cohort of MC patients, an elevated mRNA level of *TWIST1* was also found to be negatively correlated with prognosis of patients with ER+ MC [76]. We have also observed increased expression of SNAIL, and TWIST1 in ER+ MC cells consequent to forced expression of TFF3 (unpublished data). In addition to these classical molecular determinants of the EMT process, we have further observed that TFF3 stimulated increased expression of VIM (Figure 3) and SLUG (unpublished data) in ER+ MC cells. Consistent with our findings herein, forced expression of VIM in non-invasive MCF7 cells has previously been demonstrated to result in increased motility and invasiveness [77]. Elevated levels of VIM and SLUG have been also shown to be correlated with the EMT process; and also to poorer prognosis and tumour recurrence [78]. Although, EMT and cellular invasion is generally considered to be correlated with the basal-phenotype in MC [79]; we have herein provided substantial evidence that TFF3 promotes EMT and further metastatic spread of ER+ MC cells. The present findings therefore raise the possibility that TFF3 may play a critical role in the luminal progenitor compartment, which is considered to be the origin of basal-like MC [80]. Future investigations elucidating the functional role of TFF3 in the luminal progenitor compartment are thereby warranted.

## Conclusions

In summary, we have demonstrated that TFF3 functionally promotes metastatic seeding of ER+ MC cells. Forced expression of TFF3 in ER+ MC cells promotes a mesenchymal and invasive phenotype. TFF3 stimulated an invasive phenotype in ER+ MC cell through c-SRC-STAT3 mediated repression of CDH1. Furthermore, TFF3 expression predicts metastasis and poor survival outcome of patients with MC. The current findings are therapeutically relevant as early metastatic dissemination and late relapse represents a significant threat to long-term survival of patients with ER+ MC. Inhibition of TFF3 function in MC warrants consideration.

## Authors' contributions

PEL designed the research, analyzed the data, and wrote the manuscript. VP designed the research, conducted the experiments, analyzed the data, and wrote the manuscript. ZSW conducted the experiments and analyzed the data. JZ, RL, and MZ conducted the experiments and TZ analyzed the data. All authors have read and approved the manuscript for publication.

## Additional files

## Electronic supplementary material

Additional file 1: (A) Histopathological scoring. (B) qPCR primer sequence.(PDF 96 KB)

Additional file 2: **Forced expression of TFF3 in MC cells stimulates invasion.** **(A)** mRNA levels of TFF3 in various MC cell lines derived from the Oncomine database (http://www.oncomine.org) and previously reported [26]. Cell lines were subcategorised as ER- and ER+. **(B and C)** Migration and invasion of MCF7 and T47D cells, with either forced or depleted expression of TFF3, was determined by Transwell chamber assay. **(D)** Wound-healing assay, wounded areas were examined under X100 magnification using a phase contrast microscope. **(E)** Invasive capacity of MCF7 and T47D cells with either forced or depleted expression of TFF3 on exposure to vehicle (DMSO) and/or transiently transfected with control plasmids, was determined by Transwell chamber assay. Statistical significance was assessed by using an unpaired two-tailed Student's *t* test (*P <0.05* was considered as significant) using GraphPad Prism 5. Columns are the mean of triplicate experiments; bars, ± SD. ^**^P <0.001, ^*^P <0.05. (PDF 367 KB)

Additional file 3: **Forced expression of TFF3 in T47D cells modulates the mRNA levels of epithelial, mesenchymal and metastatic-related gene markers.** **(A)** qPCR analyses of T47D cells with either forced or depleted expression of TFF3 for mRNA levels of key genes functionally involved in migration, invasion, metastasis, and EMT. Change in gene expression is expressed as fold difference, respectively. Fold change values are representative of three independent biological experiments. Statistical significance was assessed by using an unpaired two-tailed Student's *t* test (*P <0.05* was considered as significant) using GraphPad Prism 5. **(B)** Western blot analysis was used to assess the protein levels of epithelial and mesenchymal markers in T47D cells with either forced or depleted expression of TFF3 as described in Methods. **(C)** Confocal microscopic visualisation of CDH1 expression in MCF7 and T47D cells with forced expression of TFF3 after exposure to JSI-124 (0.2 μM) or Stattic (2 μM). The white colour indicates CDH1 expression, and blue colour indicates nuclei stained with DAPI. Images were captured under oil immersion X600 magnification. **(D)** Confocal microscopic visualisation of VIM expression in T47D cells with either forced or depleted expression of TFF3. The red colour indicates VIM expression, and blue colour indicates nuclei stained with DAPI. Images were captured under oil immersion X600 magnification. **(E)**. Visualization of CDH1 expression in T47D cells with siRNA-mediated depleted expression of TFF3. The white colour indicates CDH1 expression, and blue colour indicates nuclei stained with DAPI. Images were captured under oil immersion X600 magnification. (PDF 430 KB)

Additional file 4: **Forced expression of TFF3 in T47D cells enhanced invasive phenotype.** **(A)** Confocal microscopic visualisation of f-actin arrangement in T47D cells with either forced or depleted expression of TFF3. The red colour indicates f-actin. Images were captured under X200 magnification. (**B)** Distribution of compact, loose, and scattered colonies of T47D cells with either forced or depleted expression of TFF3 as described in Methods. **Right side**, illustrative images of compact, loose, and scattered monolayer adherent colonies of T47D, with either forced or depleted expression of TFF3. (**C)** Capacity of T47D cells with either forced or depleted expression of TFF3 to adhere to a Collagen I matrix. **(D)** Morphology of T47D cells with either forced or depleted expression of TFF3 when cultured on a Collagen I matrix. Statistical significance was assessed by using an unpaired two-tailed Student's *t* test (*P <0.05* was considered as significant) using GraphPad Prism 5. Columns or points are the mean of triplicate experiments; bars, ± SD. ^**^P <0.001, ^*^P <0.05. (PDF 193 KB)

Additional file 5: **Forced expression of TFF3 in MC cells stimulated growth on two dimensional Matrigel; and forced expression of TFF3 in T47D cells enhanced adherence to collagen I and endothelial cells; and transmigration through an endothelial cell layer.** **(A)** Cell viability and morphology (below) of MCF7 (left side) and T47D (right side) cells with either forced or depleted expression of TFF3 when cultured on Matrigel-coated (two dimensional) plates. Images were captured under X200 magnification using phase-contrast microscopy. **(B)** Capacity of T47D cells with either forced or depleted expression of TFF3 to adhere to HMEC-1 cells. The green colour indicates T47D cells (either forced or depleted expression of TFF3), and the red colour indicates HMEC-1 cells (down side). Images were captured under X100 magnification using a fluorescence microscope, as described in Methods. **(C)**. Capacity of T47D cells with either forced or depleted expression of TFF3 to transmigrate through a HMEC-1 monolayer, as described in Methods. Statistical significance was assessed by using an unpaired two-tailed Student's *t* test (*P <*0.05 was considered as significant) using GraphPad Prism 5. Columns or points are the mean of triplicate experiments; bars, ± SD. ^**^P <0.001, ^*^P <0.05. (PDF 208 KB)

Additional file 6: **Repression of CDH1 expression is required for TFF3-stimulated invasion of MC cells.** **(A)** Effect of forced expression of *CDH1* on the MCF7 and T47D cell invasion with either forced or depleted expression of TFF3 was evaluated using a Transwell assay. Statistical significance was assessed by using an unpaired two-tailed Student's *t* test (*P <*0.05 was considered as significant) using GraphPad Prism 5. Columns are the mean of triplicate experiments; bars, ± SD. ^**^P <0.001, ^*^P <0.05. (PDF 10 KB)

Additional file 7: **Forced expression of TFF3 in T47D cells stimulated phosphorylation of STAT3 to promote invasion.** **(A)** Western blot analysis was used to assess the levels of pSTAT3, and STAT3 in T47D cells with either forced or depleted expression of TFF3 as described in Methods. **(B)** STAT3 mediated transcription, *α2-M* promoter activity in T47D cells with either forced or depleted expression of TFF3 on exposure to JSI-124 (0.2 μM) or Stattic (2 μM); and/or transiently transfected with *STAT3 DN*, *STAT3-siRNA* or *STAT3 CA*. The luciferase assay was performed as described in Methods. **(C)** *CDH1* promoter activity in T47D cells with either forced or depleted expression of TFF3 on exposure to JSI-124 (0.2 μM) or Stattic (2 μM). The luciferase assay was performed as described in Methods. **(D)** Western blot analysis was used to assess the levels of CDH1 in T47D cells with forced expression of TFF3 on exposure to JSI-124 (0.2 μM) or Stattic (2 μM) inhibitor as described in Methods. **(E)** Invasive capacity of T47D cells with either forced or depleted expression of TFF3 on exposure to JSI-124 (0.2 μM) or Stattic (2 μM); and/or transiently transfected *STAT3 DN*, *STAT3-siRNA* or *STAT3 CA*. Cell invasion was evaluated using a Transwell assay. Statistical significance was assessed by using an unpaired two-tailed Student's *t* test (*P <*0.05 was considered as significant) using GraphPad Prism 5. Columns are the mean of triplicate experiments; bars, ± SD. ^**^P <0.001, ^*^P <0.05. (PDF 74 KB)

Additional file 8: **Forced expression of TFF3 in T47D cells stimulated phosphorylation of c-SRC that subsequently increased STAT3 activity to promote invasion.** **(A)** Western blot analysis was used to assess the levels of p-c-SRC and c-SRC in T47D cells with either forced or depleted expression of TFF3. **(B)** Western blot analysis was used to assess the protein levels of pSTAT3, STAT3, and CDH1 in T47D cells with forced expression of TFF3 on exposure to PP1 (5 μM), PP2 (2 μM) or PP3 (50 μM as described in Methods. **(C)** CDH1 promoter activity in T47D cells with either forced or depleted expression of TFF3, on exposure to PP1 (5 μM), PP2 (2 μM) or PP3 (50 μM). The luciferase assay was performed as described in Methods. **(D)** STAT3 mediated transcription, α2-M promoter activity in T47D cells with either forced or depleted expression of TFF3, on exposure to PP1 (5 μM), PP2 (2 μM) or PP3 (50 μM). The luciferase assay was performed as described in Methods. **(E)** Invasive capacity of T47D cells with either forced or depleted expression of TFF3, on exposure to PP1 (5 μM), PP2 (2 μM) or PP3 (50 μM); and/or in combination with transiently transfected STAT3 CA. Invasion was evaluated using a Transwell assay. Statistical significance was assessed by using an unpaired two-tailed Student's *t* test (*P* <0.05 was considered as significant) using GraphPad Prism 5. Columns are the mean of triplicate experiments; bars, ± SD. ^**^P <0.001, ^*^P <0.05. (PDF 116 KB)

Below are the links to the authors’ original submitted files for images.

Authors’ original file for figure 1

Authors’ original file for figure 2

Authors’ original file for figure 3

Authors’ original file for figure 4

Authors’ original file for figure 5

Authors’ original file for figure 6

Authors’ original file for figure 7

## Abbreviations

BMD: benign mammary disease
CA: constitutively active
CDH1: E-cadherin
CDH2: N-cadherin
CSC: cancer stem-like cells
c-SRC: v-src avian sarcoma (Schmidt-Ruppin A-2) viral oncogene homologue
CTNNG: γ-catenin
DMSO: dimethyl sulfoxide
DN: dominant negative
EMT: epithelial-mesenchymal transition
ER: estrogen receptor
ERBB2: v-erb-b2 erythroblastic leukemia viral oncogene homologue 2
f-actin: filamentous actin
FBS: fetal bovine serum
H&E: haematoxylin and eosin
HER2: human epidermal growth factor receptor type 2
*hHPRT*: human hypoxanthine-guanine phosphoribosyltransferase
HMEC-1: human microvascular endothelial cells
IF: immunofluorescence
IgG: immunoglobulin G
IHC: immunohistochemistry
ITGA6: integrin-α6
MC: mammary carcinoma
*mgapdh*: mouse glyceraldehyde 3-phosphate dehydrogenase
OCLN: occludin
OS: overall survival
PBS: phosphate-buffered saline
PR: progesterone receptor
qPCR: quantitative PCR
RFS: relapse-free survival
siRNA: small interfering RNA
STAT3: signal transducer and activator of transcription 3
TFF3: trefoil factor 3
TMA: tissue microarray
VIM: vimentin
