# Supplementary material for: Trefoil factor 3 promotes metastatic seeding and predicts poor survival outcome of patients with mammary carcinoma
Source: Breast Cancer Res. 2014 Sep 30;16:429. doi: 10.1186/s13058-014-0429-3 (PMC4303111; doi:10.1186/s13058-014-0429-3)
Supplement: Supplementary file 1 — Additional file 1: (A) Histopathological scoring. (B) qPCR primer sequence.(PDF 96 KB) [file 13058_2014_429_MOESM1_ESM.pdf]

Additional file 1B: qPCR primer sequence

Gene		Sequence	Amplicon size
CDH1	Forward	CGAGAGCTACACGTTCACGG	119 bp
	Reverse	GGGTGTCGAGGGAAAAATAGG	
CDH2	Forward	AGCCAACCTTAACTGAGGAGT	136 bp
	Reverse	GGCAAGTTGATTGGAGGGATG	
CTNNA1	Forward	CCATGCAGGCAACATAAACTTC	81 bp
	Reverse	GGCTCCAACAGTCTCTCAACT	
CTNNB1	Forward	CCCACTGGCCTCTGATAAAGG	80 bp
	Reverse	ACGCAAAGGTGCATGATTTG	
CTNND1	Forward	TTCATCACAGGTGCTGCGTAA	93 bp
	Reverse	CCATCACACTCTCTCATCCTTCTG	
FN1	Forward	GGTGACACTTATGAGCGTCCTAAA	170 bp
	Reverse	AACATGTAACCACCAGTCTCATGTG	
FOXC2	Forward	CCTCCTGGTATCTCAACCACA	131 bp
	Reverse	GGTCGAGTTCTCAATCCCCA	
MET	Forward	TGGTGCAGAGGAGCAATGG	111 bp
	Reverse	CATTCTGGATGGGTGTTTCCG	
MMP2	Forward	CAAAAACAAGAAGACATACATCTT	232 bp
	Reverse	GCTTCCAAACTTCACGCTC	
MTA1	Forward	GCTGTTACACCACACAGTCTT	166 bp
	Reverse	GGACTCATGTTACTGCGGTTT	
MTA2	Forward	CCGACGGCCTTATGCTCCT	145 bp
	Reverse	CTGGGCCACCAGATCTTTGAC	
NME1	Forward	CTGCAGCCGGAGTTCAAAC	68 bp
	Reverse	GCAATGAAGGTACGCTCACAGT	
OCLN	Forward	TGCCGCGTTGGTGATCTTT	100 bp
	Reverse	GCCCAGGATAGCACTCACTATT	
PLAU	Forward	CACGCAAGGGGAGATGAA	341 bp
	Reverse	ACAGCATTTTGGTGGTGACTT	
PLAUR	Forward	AATGGCCGCCAGTGTTACAG	227 bp
	Reverse	CAGGAGACATCAATGTGGTTC	
SERPINB5	Forward	CTACTTTGTTGGCAAGTGGATGAA	90 bp
	Reverse	ACTGGTTTGGTGTCTGTCTTGTTG	
TIMP1	Forward	CTTCTGGCATCCTGTTGTTG	84 bp
	Reverse	AGAAGGCCGTCTGTGGGT	
VIM	Forward	CCTTGAACGCAAAGTGGAATC	106 bp
	Reverse	GACATGCTGTTCCTGAATCTGAG	
