# Supplementary figures and images for: Trefoil factor 3 promotes metastatic seeding and predicts poor survival outcome of patients with mammary carcinoma
Source: Breast Cancer Res. 2014 Sep 30;16:429. doi: 10.1186/s13058-014-0429-3 (PMC4303111; doi:10.1186/s13058-014-0429-3)

Additional file 2

A. TFF3 mRNA levels

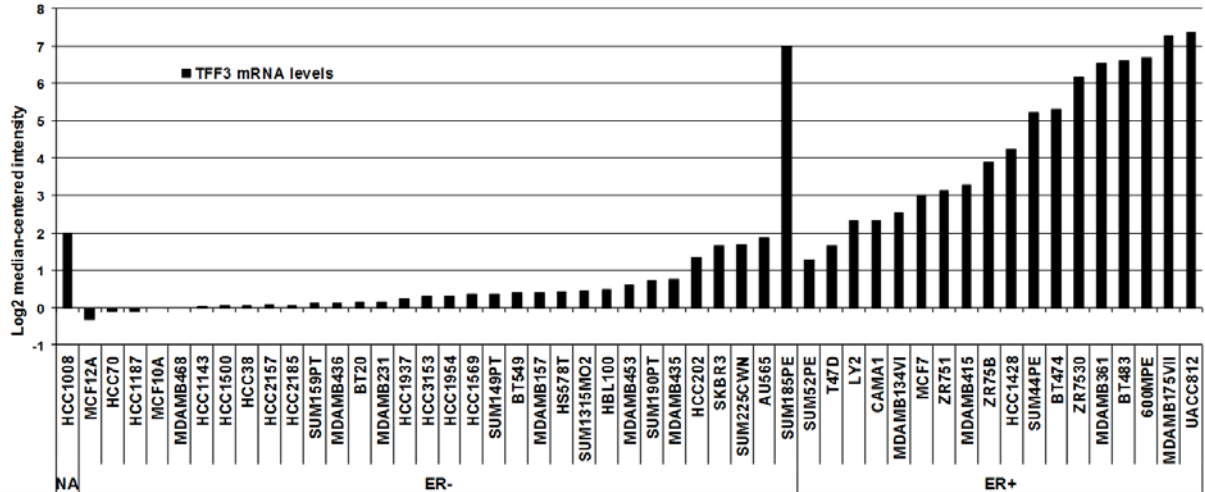

B. Migration

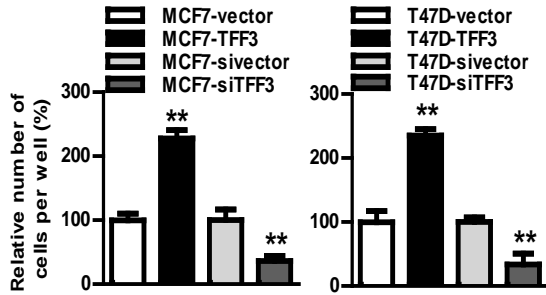

C. Invasion

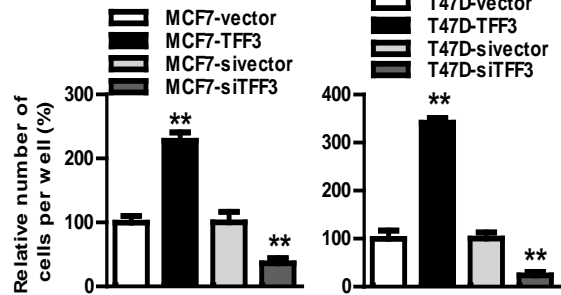

D. Wound healing

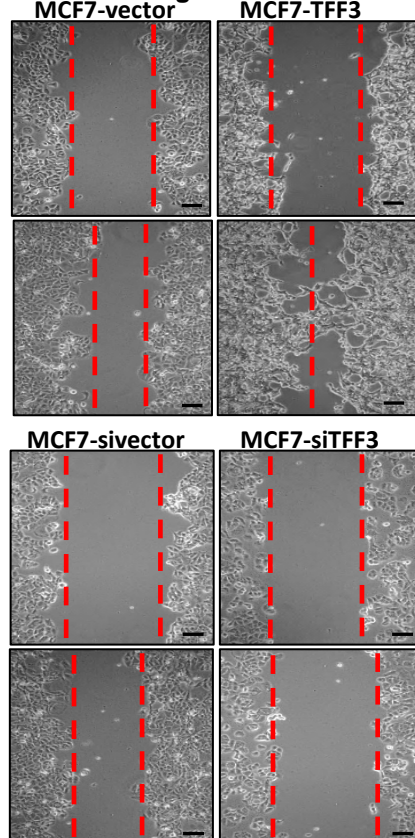

E. Invasion

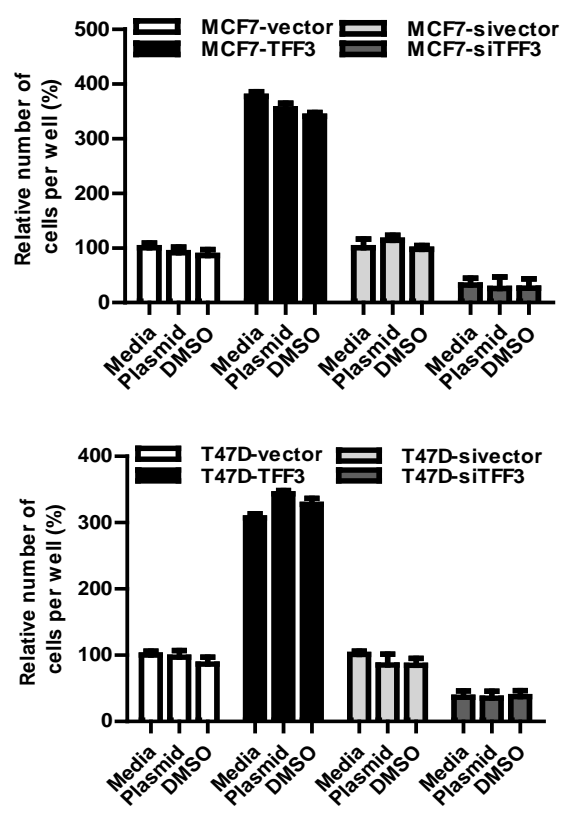

Supplement: Supplementary file 2 — Additional file 2: Forced expression of TFF3 in MC cells stimulates invasion. (A) mRNA levels of TFF3 in various MC cell lines derived from the Oncomine database (http://www.oncomine.org) and previously reported [26]. Cell lines were subcategorised as ER- and ER+. (B and C) Migration and invasion of MCF7 and T47D cells, with either forced or depleted expression of TFF3, was determined by Transwell chamber assay. (D) Wound-healing assay, wounded areas were examined under X100 magnification using a phase contrast microscope. (E) Invasive capacity of MCF7 and T47D cells with either forced or depleted expression of TFF3 on exposure to vehicle (DMSO) and/or transiently transfected with control plasmids, was determined by Transwell chamber assay. Statistical significance was assessed by using an unpaired two-tailed Student's t test (P <0.05 was considered as significant) using GraphPad Prism 5. Columns are the mean of triplicate experiments; bars, ± SD. **P <0.001, *P <0.05. (PDF 367 KB) [file 13058_2014_429_MOESM2_ESM.pdf]

Additional file 4

A. f-actin

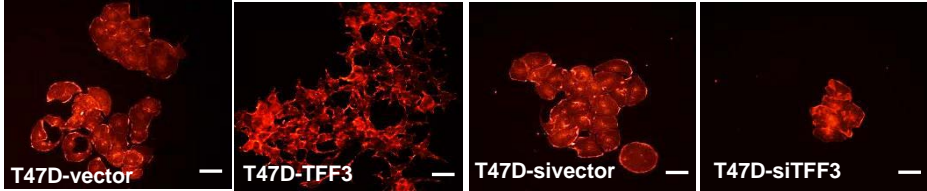

B. Colony scattering

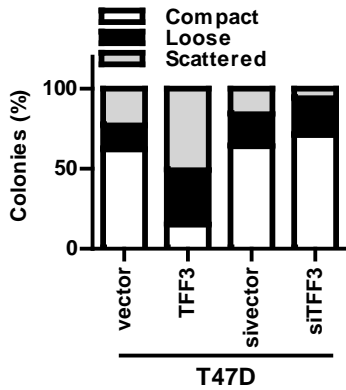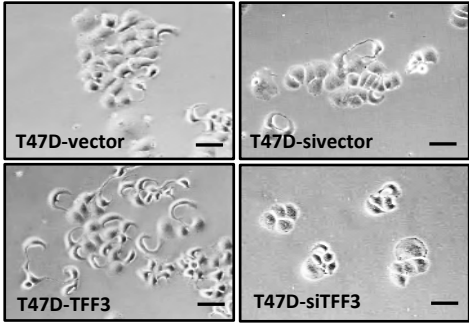

C. Collagen I adhesion

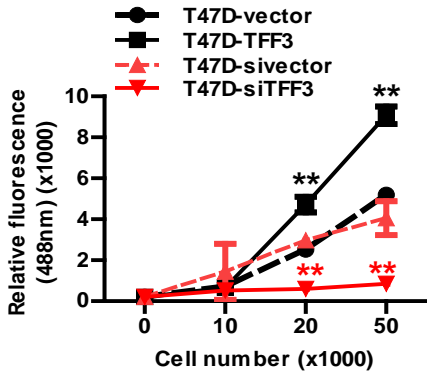

D. Collagen I

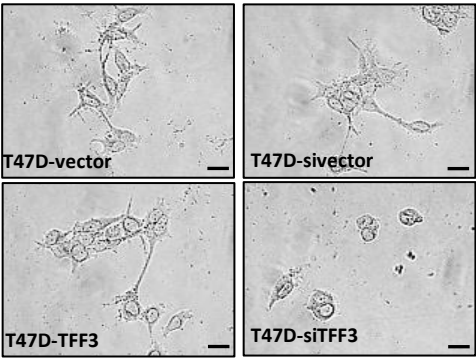

Supplement: Supplementary file 4 — Additional file 4: Forced expression of TFF3 in T47D cells enhanced invasive phenotype. (A) Confocal microscopic visualisation of f-actin arrangement in T47D cells with either forced or depleted expression of TFF3. The red colour indicates f-actin. Images were captured under X200 magnification. (B) Distribution of compact, loose, and scattered colonies of T47D cells with either forced or depleted expression of TFF3 as described in Methods. Right side, illustrative images of compact, loose, and scattered monolayer adherent colonies of T47D, with either forced or depleted expression of TFF3. (C) Capacity of T47D cells with either forced or depleted expression of TFF3 to adhere to a Collagen I matrix. (D) Morphology of T47D cells with either forced or depleted expression of TFF3 when cultured on a Collagen I matrix. Statistical significance was assessed by using an unpaired two-tailed Student's t test (P <0.05 was considered as significant) using GraphPad Prism 5. Columns or points are the mean of triplicate experiments; bars, ± SD. **P <0.001, *P <0.05. (PDF 193 KB) [file 13058_2014_429_MOESM4_ESM.pdf]

Additional file 5

A. 2D Matrigel

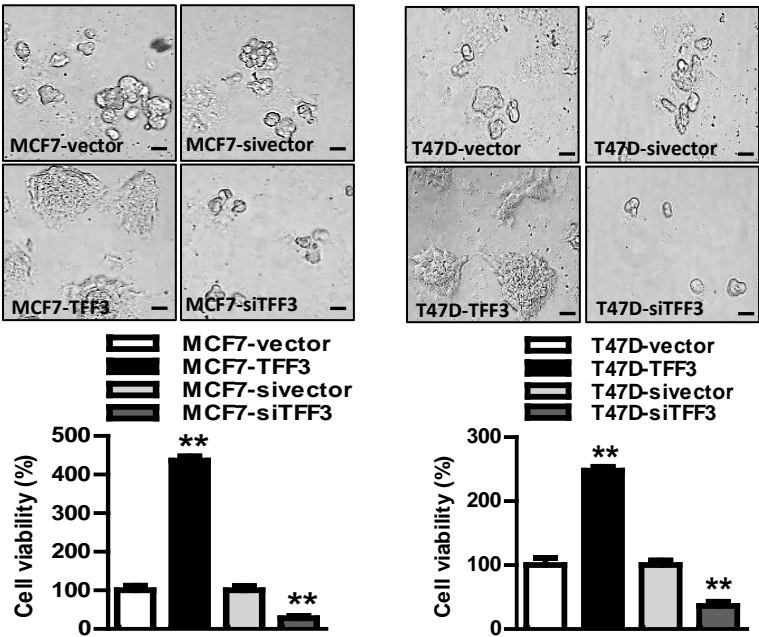

B. Endothelial cell adhesion

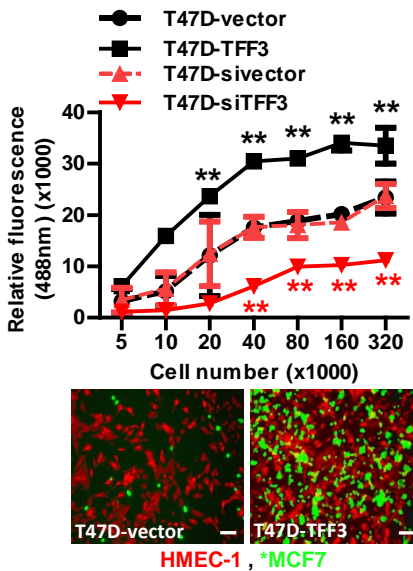

C. Endothelial transmigration

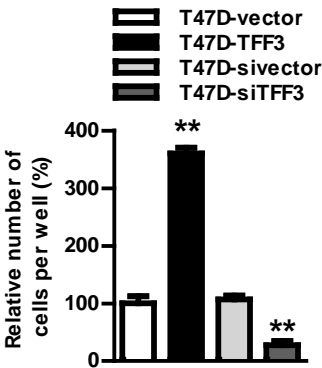

Supplement: Supplementary file 5 — Additional file 5: Forced expression of TFF3 in MC cells stimulated growth on two dimensional Matrigel; and forced expression of TFF3 in T47D cells enhanced adherence to collagen I and endothelial cells; and transmigration through an endothelial cell layer. (A) Cell viability and morphology (below) of MCF7 (left side) and T47D (right side) cells with either forced or depleted expression of TFF3 when cultured on Matrigel-coated (two dimensional) plates. Images were captured under X200 magnification using phase-contrast microscopy. (B) Capacity of T47D cells with either forced or depleted expression of TFF3 to adhere to HMEC-1 cells. The green colour indicates T47D cells (either forced or depleted expression of TFF3), and the red colour indicates HMEC-1 cells (down side). Images were captured under X100 magnification using a fluorescence microscope, as described in Methods. (C). Capacity of T47D cells with either forced or depleted expression of TFF3 to transmigrate through a HMEC-1 monolayer, as described in Methods. Statistical significance was assessed by using an unpaired two-tailed Student's t test (P <0.05 was considered as significant) using GraphPad Prism 5. Columns or points are the mean of triplicate experiments; bars, ± SD. **P <0.001, *P <0.05. (PDF 208 KB) [file 13058_2014_429_MOESM5_ESM.pdf]

Additional file 6

A. Invasion

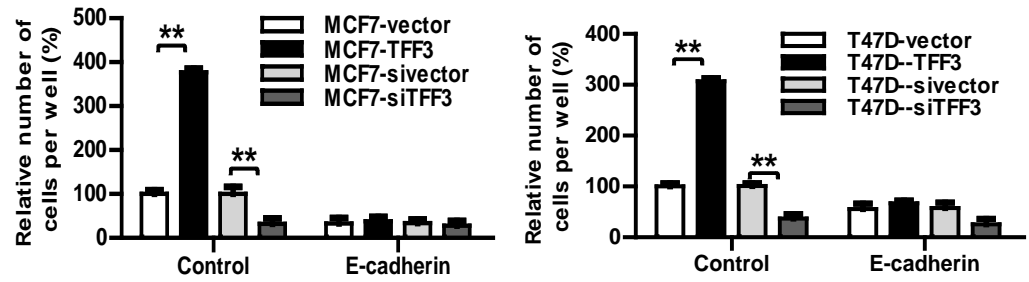

Supplement: Supplementary file 6 — Additional file 6: Repression of CDH1 expression is required for TFF3-stimulated invasion of MC cells. (A) Effect of forced expression of CDH1 on the MCF7 and T47D cell invasion with either forced or depleted expression of TFF3 was evaluated using a Transwell assay. Statistical significance was assessed by using an unpaired two-tailed Student's t test (P <0.05 was considered as significant) using GraphPad Prism 5. Columns are the mean of triplicate experiments; bars, ± SD. **P <0.001, *P <0.05. (PDF 10 KB) [file 13058_2014_429_MOESM6_ESM.pdf]

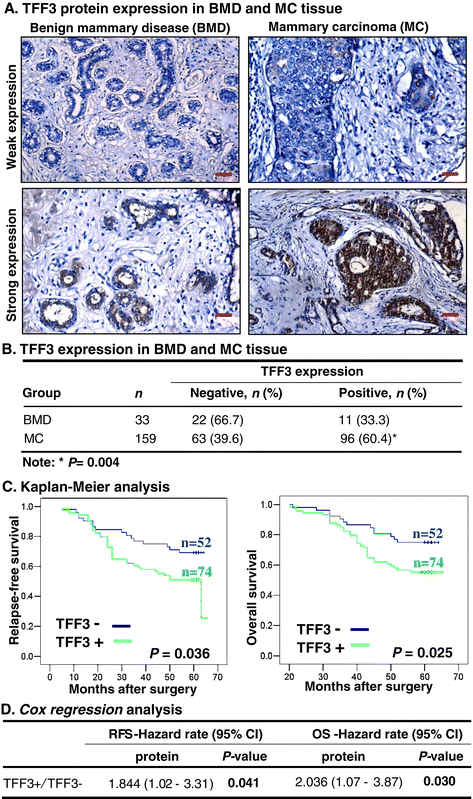

Supplement: Supplementary file 9 — Authors’ original file for figure 1 [file 13058_2014_429_MOESM9_ESM.gif]

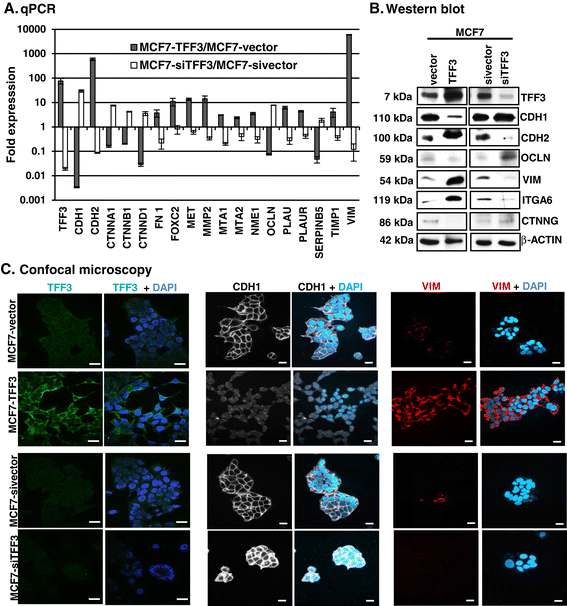

Supplement: Supplementary file 10 — Authors’ original file for figure 2 [file 13058_2014_429_MOESM10_ESM.gif]

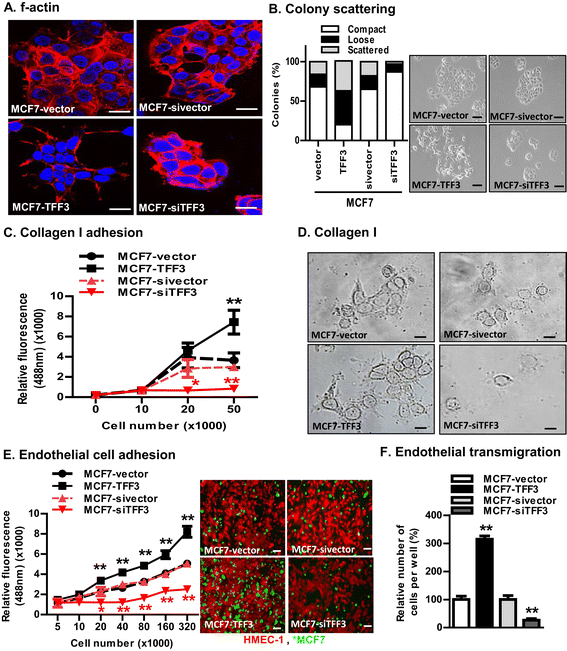

Supplement: Supplementary file 11 — Authors’ original file for figure 3 [file 13058_2014_429_MOESM11_ESM.gif]

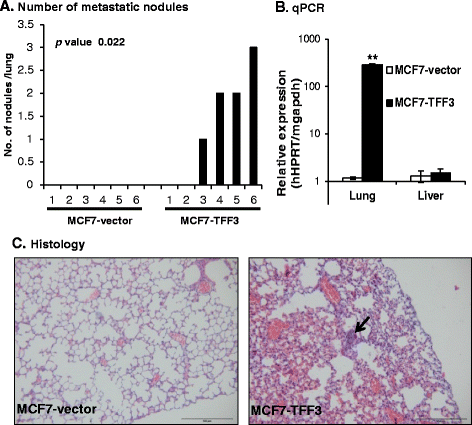

Supplement: Supplementary file 12 — Authors’ original file for figure 4 [file 13058_2014_429_MOESM12_ESM.gif]

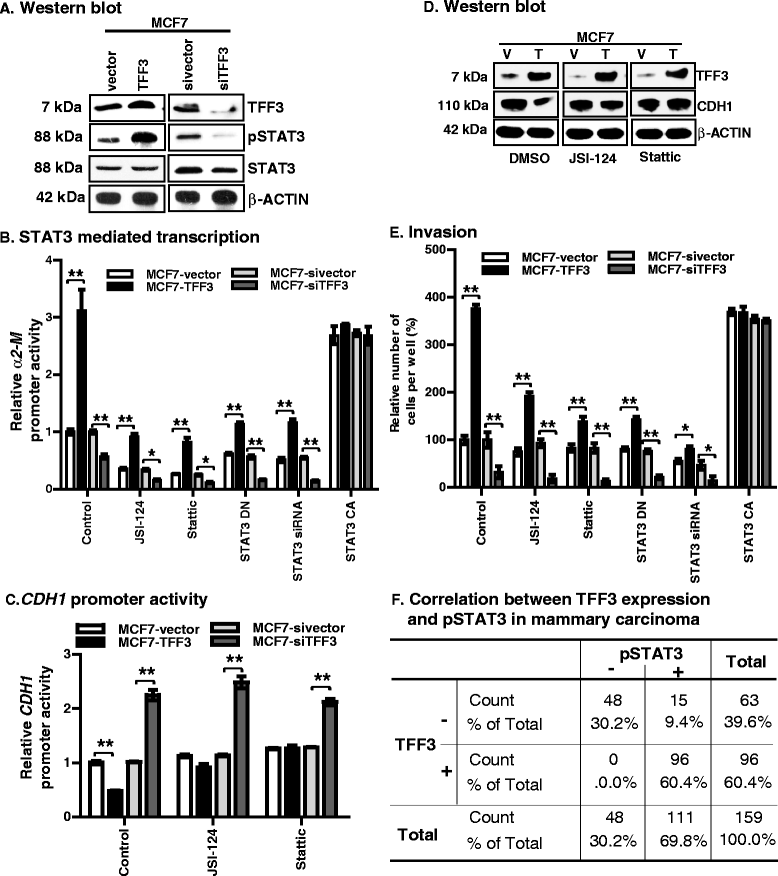

Supplement: Supplementary file 13 — Authors’ original file for figure 5 [file 13058_2014_429_MOESM13_ESM.gif]

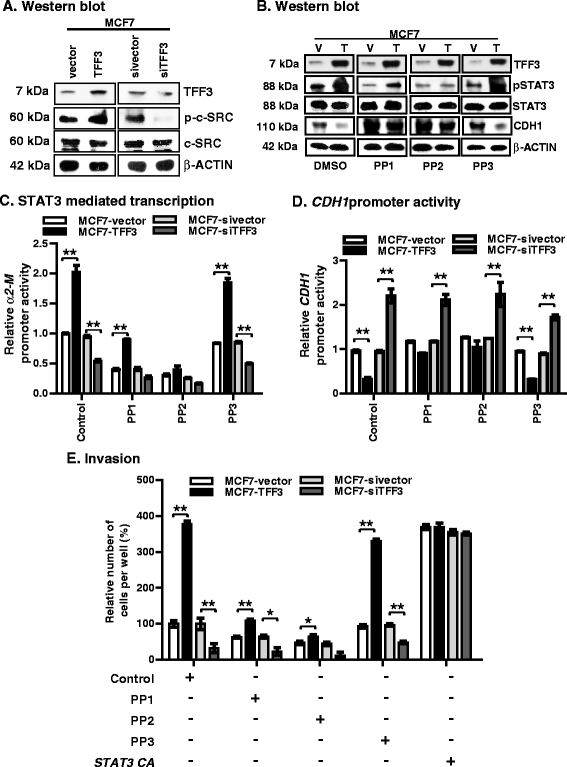

Supplement: Supplementary file 14 — Authors’ original file for figure 6 [file 13058_2014_429_MOESM14_ESM.gif]
