# Supplementary material for: Trefoil factor 3 promotes metastatic seeding and predicts poor survival outcome of patients with mammary carcinoma
Source: Breast Cancer Res. 2014 Sep 30;16:429. doi: 10.1186/s13058-014-0429-3 (PMC4303111; doi:10.1186/s13058-014-0429-3)

Additional file 3

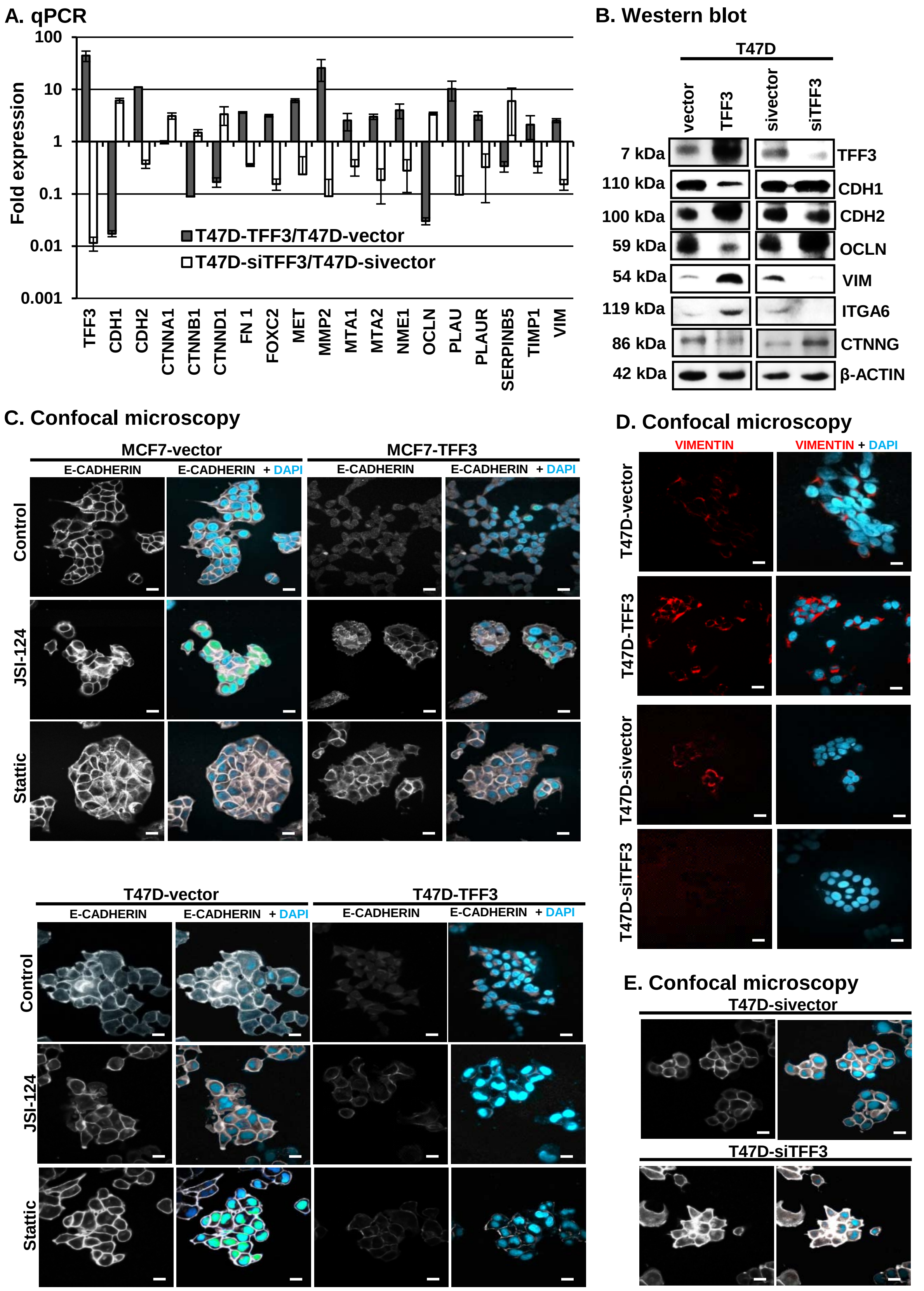

Supplement: Supplementary file 3 — Additional file 3: Forced expression of TFF3 in T47D cells modulates the mRNA levels of epithelial, mesenchymal and metastatic-related gene markers. (A) qPCR analyses of T47D cells with either forced or depleted expression of TFF3 for mRNA levels of key genes functionally involved in migration, invasion, metastasis, and EMT. Change in gene expression is expressed as fold difference, respectively. Fold change values are representative of three independent biological experiments. Statistical significance was assessed by using an unpaired two-tailed Student's t test (P <0.05 was considered as significant) using GraphPad Prism 5. (B) Western blot analysis was used to assess the protein levels of epithelial and mesenchymal markers in T47D cells with either forced or depleted expression of TFF3 as described in Methods. (C) Confocal microscopic visualisation of CDH1 expression in MCF7 and T47D cells with forced expression of TFF3 after exposure to JSI-124 (0.2 μM) or Stattic (2 μM). The white colour indicates CDH1 expression, and blue colour indicates nuclei stained with DAPI. Images were captured under oil immersion X600 magnification. (D) Confocal microscopic visualisation of VIM expression in T47D cells with either forced or depleted expression of TFF3. The red colour indicates VIM expression, and blue colour indicates nuclei stained with DAPI. Images were captured under oil immersion X600 magnification. (E). Visualization of CDH1 expression in T47D cells with siRNA-mediated depleted expression of TFF3. The white colour indicates CDH1 expression, and blue colour indicates nuclei stained with DAPI. Images were captured under oil immersion X600 magnification. (PDF 430 KB) [file 13058_2014_429_MOESM3_ESM.pdf]
