# Supplementary material for: Trefoil factor 3 promotes metastatic seeding and predicts poor survival outcome of patients with mammary carcinoma
Source: Breast Cancer Res. 2014 Sep 30;16:429. doi: 10.1186/s13058-014-0429-3 (PMC4303111; doi:10.1186/s13058-014-0429-3)

Additional file 7

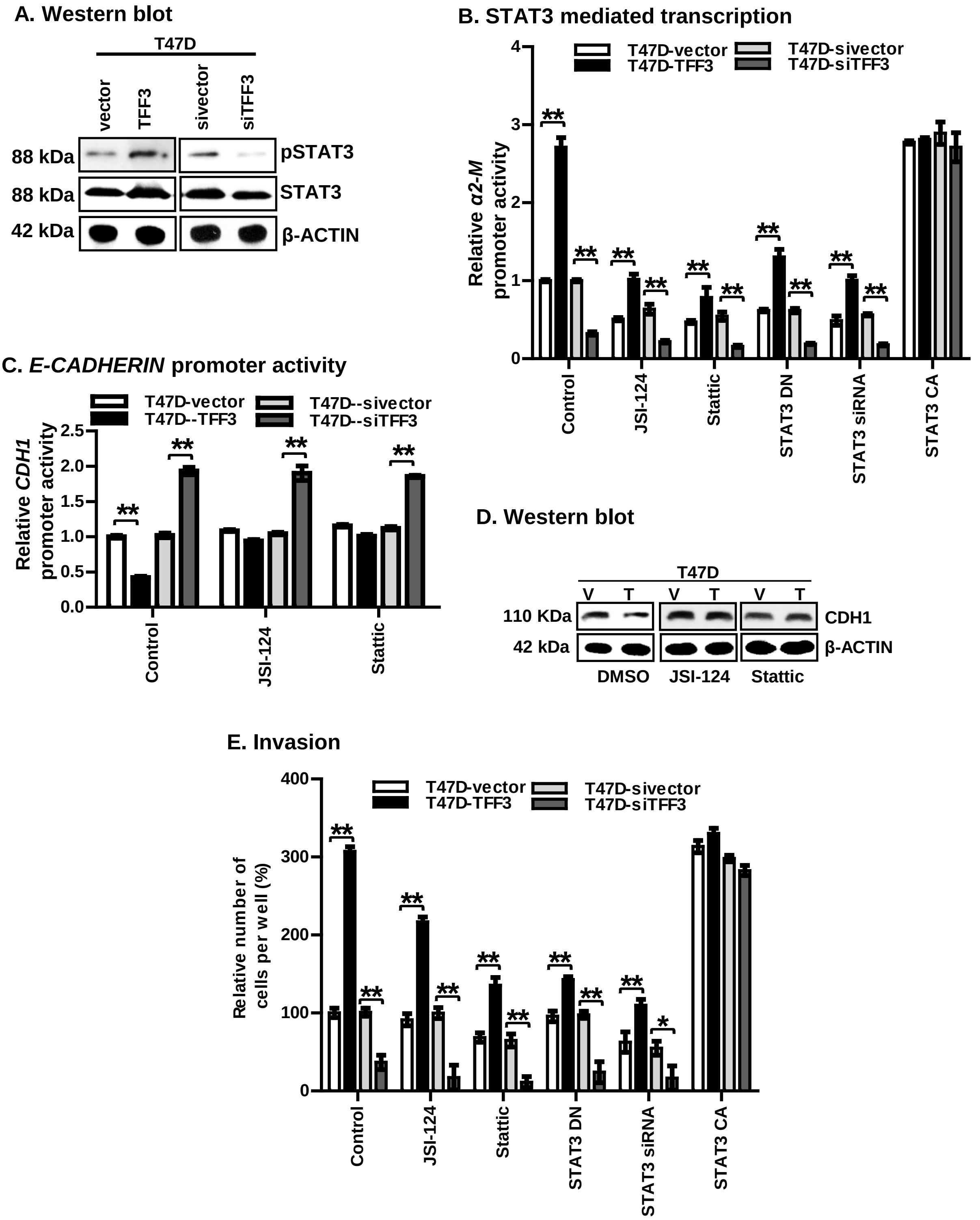

Supplement: Supplementary file 7 — Additional file 7: Forced expression of TFF3 in T47D cells stimulated phosphorylation of STAT3 to promote invasion. (A) Western blot analysis was used to assess the levels of pSTAT3, and STAT3 in T47D cells with either forced or depleted expression of TFF3 as described in Methods. (B) STAT3 mediated transcription, α2-M promoter activity in T47D cells with either forced or depleted expression of TFF3 on exposure to JSI-124 (0.2 μM) or Stattic (2 μM); and/or transiently transfected with STAT3 DN, STAT3-siRNA or STAT3 CA. The luciferase assay was performed as described in Methods. (C) CDH1 promoter activity in T47D cells with either forced or depleted expression of TFF3 on exposure to JSI-124 (0.2 μM) or Stattic (2 μM). The luciferase assay was performed as described in Methods. (D) Western blot analysis was used to assess the levels of CDH1 in T47D cells with forced expression of TFF3 on exposure to JSI-124 (0.2 μM) or Stattic (2 μM) inhibitor as described in Methods. (E) Invasive capacity of T47D cells with either forced or depleted expression of TFF3 on exposure to JSI-124 (0.2 μM) or Stattic (2 μM); and/or transiently transfected STAT3 DN, STAT3-siRNA or STAT3 CA. Cell invasion was evaluated using a Transwell assay. Statistical significance was assessed by using an unpaired two-tailed Student's t test (P <0.05 was considered as significant) using GraphPad Prism 5. Columns are the mean of triplicate experiments; bars, ± SD. **P <0.001, *P <0.05. (PDF 74 KB) [file 13058_2014_429_MOESM7_ESM.pdf]
