# Supplementary material for: Trefoil factor 3 promotes metastatic seeding and predicts poor survival outcome of patients with mammary carcinoma
Source: Breast Cancer Res. 2014 Sep 30;16:429. doi: 10.1186/s13058-014-0429-3 (PMC4303111; doi:10.1186/s13058-014-0429-3)

Additional file 8

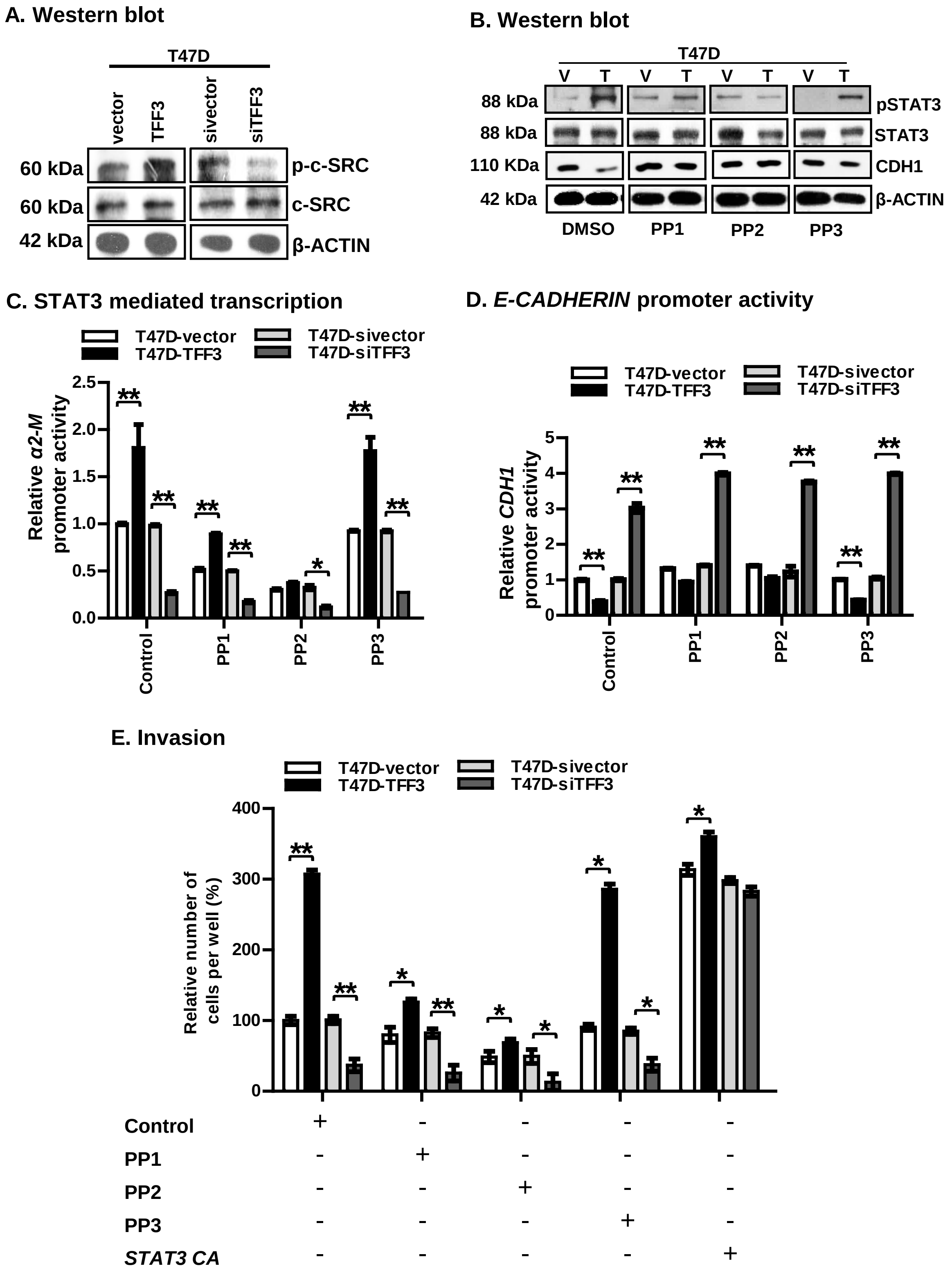

Supplement: Supplementary file 8 — Additional file 8: Forced expression of TFF3 in T47D cells stimulated phosphorylation of c-SRC that subsequently increased STAT3 activity to promote invasion. (A) Western blot analysis was used to assess the levels of p-c-SRC and c-SRC in T47D cells with either forced or depleted expression of TFF3. (B) Western blot analysis was used to assess the protein levels of pSTAT3, STAT3, and CDH1 in T47D cells with forced expression of TFF3 on exposure to PP1 (5 μM), PP2 (2 μM) or PP3 (50 μM as described in Methods. (C) CDH1 promoter activity in T47D cells with either forced or depleted expression of TFF3, on exposure to PP1 (5 μM), PP2 (2 μM) or PP3 (50 μM). The luciferase assay was performed as described in Methods. (D) STAT3 mediated transcription, α2-M promoter activity in T47D cells with either forced or depleted expression of TFF3, on exposure to PP1 (5 μM), PP2 (2 μM) or PP3 (50 μM). The luciferase assay was performed as described in Methods. (E) Invasive capacity of T47D cells with either forced or depleted expression of TFF3, on exposure to PP1 (5 μM), PP2 (2 μM) or PP3 (50 μM); and/or in combination with transiently transfected STAT3 CA. Invasion was evaluated using a Transwell assay. Statistical significance was assessed by using an unpaired two-tailed Student's t test (P <0.05 was considered as significant) using GraphPad Prism 5. Columns are the mean of triplicate experiments; bars, ± SD. **P <0.001, *P <0.05. (PDF 116 KB) [file 13058_2014_429_MOESM8_ESM.pdf]
