# Supplementary material for: Trefoil factor 3 promotes metastatic seeding and predicts poor survival outcome of patients with mammary carcinoma
Source: Breast Cancer Res. 2014 Sep 30;16:429. doi: 10.1186/s13058-014-0429-3 (PMC4303111; doi:10.1186/s13058-014-0429-3)
Supplement: Supplementary file 15 — Authors’ original file for figure 7 [file 13058_2014_429_MOESM15_ESM.pdf]

Table 1. Associations between TFF3 expression in MC and the clinicopathological characteristics of the tumor cohort.

**Parameter**	***n***	**TFF3 positive expression, *n* (%)**	***p value***
Age (years)			
≤ 35	16	9 (56.3)	0.939
>35-≤55	92	56 (60.9)	
> 55	51	31 (60.8)	
Tumor size (cm)			
≤ 2	13	2 (15.4)	**0.002**
>2-≤5	115	72 (62.6)	
> 5	31	22 (71.0)	
Lymph node [illegible]			
0	55	25 (45.5)	**0.004**
≤3	55	33 (60.0)	
>3	49	38 (77.6)	
Grade			
I	13	7 (53.8)	0.269
II	102	58 (56.9)	
III	44	31 (70.5)	
Stage			
I-II	85	45 (52.9)	**0.040**
III-IV	74	51 (68.9)	
ER			
−	94	54 (57.4)	0.364
+	65	42 (64.6)	
PR			
−	90	54 (60.0)	0.912
+	69	42 (60.9)	
ERBB2			
low	107	69 (64.5)	0.129
high	52	27 (51.9)	

^ ER+ required at least 10% staining nuclei.
^^PR+ required at least 10% staining nuclei.
Values in bold are significant (*P* <0.005)
